# Bladder Cancer Incidence Trends in the United States From 2000 to 2020

**DOI:** 10.1002/cnr2.70548

**Published:** 2026-04-28

**Authors:** Seyed Ehsan Mousavi, Ryan Michael Antar, Armin Aslani, Pourya Shokri, Zahra Yekta, Amin Bateni, Nasser Shakhssalim, Seyed Aria Nejadghaderi

**Affiliations:** ^1^ Neurosciences Research Center, Aging Research Institute Tabriz University of Medical Sciences Tabriz Iran; ^2^ Faculty of Medicine, Social Determinants of Health Research Center, Department of Community Medicine Tabriz University of Medical Sciences Tabriz Iran; ^3^ School of Medicine & Health Sciences The George Washington University Washington District of Columbia USA; ^4^ School of Medicine Shahid Beheshti University of Medical Sciences Tehran Iran; ^5^ Calaveras County Department of Health Calaveras County California USA; ^6^ Department of Community Medicine, School of Medicine Urmia University of Medical Sciences Urmia Iran; ^7^ Urology and Nephrology Research Center Shahid Beheshti University of Medical Sciences Tehran Iran; ^8^ HIV/STI Surveillance Research Center, and WHO Collaborating Center for HIV Surveillance, Institute for Futures Studies in Health Kerman University of Medical Sciences Kerman Iran; ^9^ Knowledge Hub for Migrant and Refugee Health, Institute for Futures Studies in Health Kerman University of Medical Sciences Kerman Iran

**Keywords:** bladder cancer, bladder neoplasm, epidemiology, incidence, SEER, surveillance, epidemiology, and end results, United States

## Abstract

**Background:**

Bladder cancer is one of the most common urogenital cancers globally. Here, we aimed to report the incidence trend of bladder cancer in the United States (US) over 2000–2020, by age, sex, race/ethnicity, and types using Surveillance, Epidemiology, and End Results (SEER).

**Methods:**

We used SEER 22 data to calculate the age‐standardized and delay‐adjustment incidence rate of bladder cancer. Only individuals diagnosed with cancer and whose age at diagnosis was known were included in the study. A delay model was applied, adjusting for variables such as cancer site, registry, age group, race/ethnicity, and year of diagnosis. Using the Tiwari technique, the same database was also used to determine the age‐standardized incidence rate (ASIR) of subtypes. The incidence data for 2020 were excluded from Joinpoint trends while displayed separately in illustrations.

**Results:**

Over 2000–2019, most bladder cancer cases were urothelial carcinoma (92.99%), among non‐Hispanic Whites (84.48%) and those aged 70 to 84 years (45.72%). There was a significant decrease in the ASIR of bladder cancer across all races/ethnicities in both sexes within all age groups (Average annual percent change (AAPC): −0.95%; [−1.06, −0.83] for men and −1.02% [−1.21, −0.84] for women) over 2000–2019. The incidence rates decreased by 6.23% during the COVID‐19 pandemic. Also, the delay‐adjusted incidence rate of bladder cancer increased with advancing age.

**Conclusions:**

Despite a decrease in ASIR of bladder cancer over 2000–2019, there are still a high number of incident cases, particularly among NHWs and the elderly. Future studies should investigate the underlying causes of bladder cancer incidence trends among demographic groups.

**Abbreviations**
AAPC: average annual percent change
APC: annual percent change
ASIR: age‐standardized incidence rate
MIBC: muscle‐invasive bladder cancer
NHB: non‐hispanic black
NHW: non‐hispanic white
SCC: squamous cell carcinoma
SEER: surveillance, epidemiology, and end results
US: United States

## 1 Introduction

Bladder cancer is among the top 10 most prevalent cancers globally, with approximately 613 791 new cases and around 220 349 deaths reported worldwide in 2022. The worldwide age‐standardized incidence rate (per 100 000 person‐years) is 9.5 in men and 2.4 in women [1]. It is more frequently diagnosed in men than women and often initiates without noticeable symptoms, painless microscopic or gross hematuria [2]. Approximately 75% of affected patients are diagnosed with non‐muscle‐invasive bladder cancer at the time of diagnosis, while the remaining 25% have muscle‐invasive bladder cancer (MIBC) [1, 3]. Urinary bladder cancer treatment options used to be limited to surgery and chemotherapy as the gold standard, with radical removal of the bladder being the preferred method for MIBC [4].

The economic burden for urinary bladder cancer in the United States (US) is estimated to be close to one billion dollars annually [5, 6]. In the US, there are approximately 84 530 estimated new cases and 17 870 deaths in 2026, with incidence rates higher among the elderly and Whites [7]. Bladder cancer is more prevalent in specific populations such as American Indian/Alaska natives [8]. Considering factors such as population growth, aging, and exposure to risk factors, it is predicted that the incident cases of bladder cancer will increase [9, 10]. On the other hand, the emergence of diseases like COVID‐19 can have an indirect impact on the diagnosis and prognosis of cancers, such as bladder cancer [11]. Due to the pandemic, screening for cancer was suspended, resulting in fewer diagnosed cases and more advanced stages [12]. As different morphological subtypes like urothelial carcinoma, adenocarcinoma, and squamous cell carcinoma have variations in response to treatment and survival rates, it is important to report the epidemiology of each subtype separately [13].

Numerous studies have been conducted on urinary bladder cancer, focusing on its classification, diagnostic methods, treatment options, incidence rate, and economic burden [8, 14]. A recent study used data from the Surveillance, Epidemiology and End Results (SEER) database and the National Center for Health Statistics to report the incidence and mortality of urogenital cancers in the US in the last decades [8]. However, existing literature often lacks a contemporary, comprehensive analysis of long‐term incidence trends that simultaneously incorporates detailed demographic stratification, morphological subtypes, and the distinct impact of the COVID‐19 pandemic on reported cases [8]. Therefore, herein, we utilized the SEER data to investigate the incidence of urinary bladder cancer in the US over the two‐decade period of 2000–2020. Our specific objectives were to: (1) Report the detailed incidence trends of overall bladder cancer by age, sex, and race/ethnicity; (2) Determine the specific age‐standardized incidence rates (ASIRs) and trends for all major morphological subtypes, including urothelial carcinoma, squamous cell carcinoma (SCC), adenocarcinoma, small cell neuroendocrine carcinoma, and sarcoma; and (3) Quantify the immediate effect of the COVID‐19 pandemic on the reported incidence rates during 2020. The findings of this study are important as they can provide updated epidemiological data on bladder cancer disparities, which can be helpful for health policymakers for resource allocation, and inform the implementation of targeted preventive measures and enhanced surveillance strategies for each high‐risk demographic group and specific histological subtype.

## 2 Methods

The SEER program, developed by the National Cancer Institute, functions as a comprehensive population‐based database for cancer‐related information in the United States. SEER 22 encompasses about 48% of the US population and provides essential data on cancer staging at diagnosis and patient survival rates. The program collects a wide range of information, including demographic details of patients, the primary site of tumors, tumor morphology, the stage at diagnosis, initial treatments, and ongoing tracking of vital status [15]. For this study, the SEER 22 database, updated as of November 2022, was employed to estimate bladder cancer incidence rates and annual percent changes (APCs) between 2000 and 2020 [16, 17]. Access to the SEER 22 database was granted under the SEER Research Data Agreement for 1975–2020 Data (November 2022 Submission) [18], and the reporting of cancer statistics adhered to SEER 22's established guidelines [19].

Bladder cancers were identified using the International Classification of Diseases for Oncology version 3 (ICD‐O‐3). Bladder cancer was classified as urothelial carcinoma (i.e., transitional cell carcinoma) (codes: 8020, 8031, 8082, 8120, 8122, 8130, and 8131), adenocarcinoma (codes: 8140, 8144, 8310, 8380, and 8480), sarcoma (codes: 8800, 8802, 8850, 8890, 8900, 8910, 9120, 9220, and 9540), small cell neuroendocrine carcinoma (codes: 8013, 8041, and 8240), and SCC (codes: 8051 and 8070).

The ASIRs were calculated [16], using SEER*Stat, version 8.4.1.2 [20]. A delay model was used, which is recommneded by the SEER program [21, 22]. This method, commonly known as delay‐adjustment, is recommended by the SEER program to account for the reporting delay of cancer cases and to ensure more accurate incidence rate estimates, particularly for the most recent years of the study period. This technique is highly appropriate for large, ongoing registry data as it mathematically corrects for the lag inherent in processing vast numbers of case reports. The same database was also used to determine the ASIR of bladder cancer subtypes using the Tiwari technique [23], which is incorporated into the SEER*Stat software for robust rate calculations. The Tiwari technique is the standard demographic method used by SEER for calculating ASIRs, allowing for valid comparisons of rates across different racial and ethnic groups and time periods within this extensive dataset. The Joinpoint Regression Program, version 5.0.2, was utilized for this analysis [24, 25]. This program is the standard tool recommended by the National Cancer Institute for analyzing long‐term cancer rates and determining the time points where the trend significantly changes. The year 2020 incidence data were excluded from Joinpoint trends and only shown in graphs, adhering to established SEER and National Cancer Institute guidelines for trend analysis, which recommend excluding the most recent year's data from regression modeling for stability [19]. Least‐squares regression lines were used to compute the bladder cancer APCs [26]. Weighted Bayesian Information Criteria approach was used foe model selection [27, 28]. The parallelism test compared trends between two groups over time, while the coincidence test determined if rates between two groups were identical over time [29].

In summary, the methodology and steps of the study were as follows: Access SEER 22 database (November 2022 submission) → Identify bladder cancer cases using ICD‐O‐3 codes for subtypes → Calculate delay‐adjusted ASIRs using SEER*Stat software with delay model and Tiwari technique → Stratify by age, sex, race/ethnicity, and subtypes → Analyze trends (2000–2019) using Joinpoint Regression Program to compute AAPCs, parallelism, and coincidence tests → Exclude 2020 from trends but display separately in figures for COVID‐19 impact assessment.

## 3 Results

### 3.1 Declining Overall Bladder Cancer Incidence Trends, With Highest Rates Among Non‐Hispanic White Males Aged 70–84 Years

**FIGURE 1:**
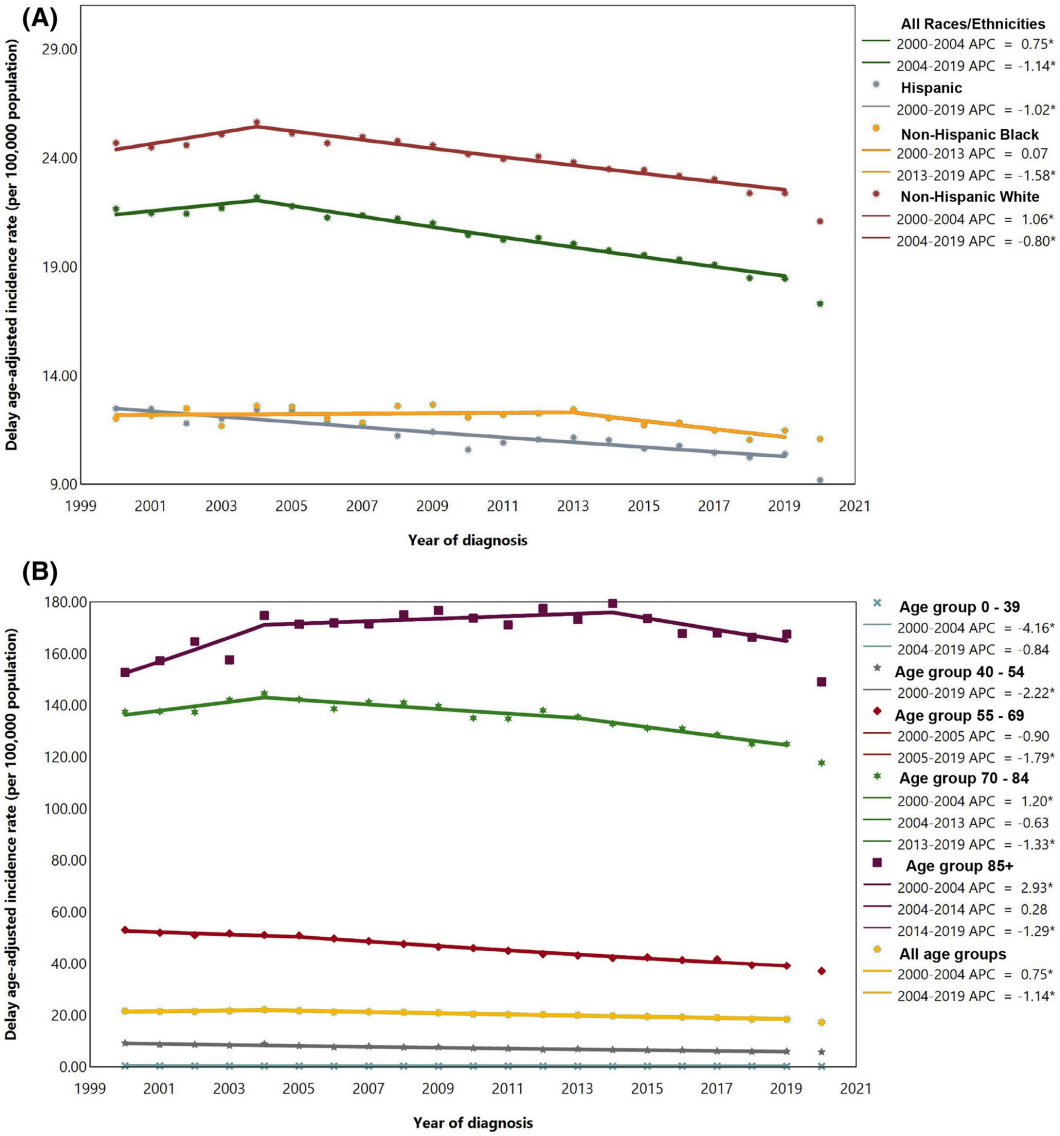
Delayed age‐adjusted incidence rate of bladder cancer over 2000–2019 and in 2020 in the United States, by race/ethnicity (A) and age (B). APC, Annual percent change. * Represent *p*‐value less than 0.05.

**FIGURE 2:**
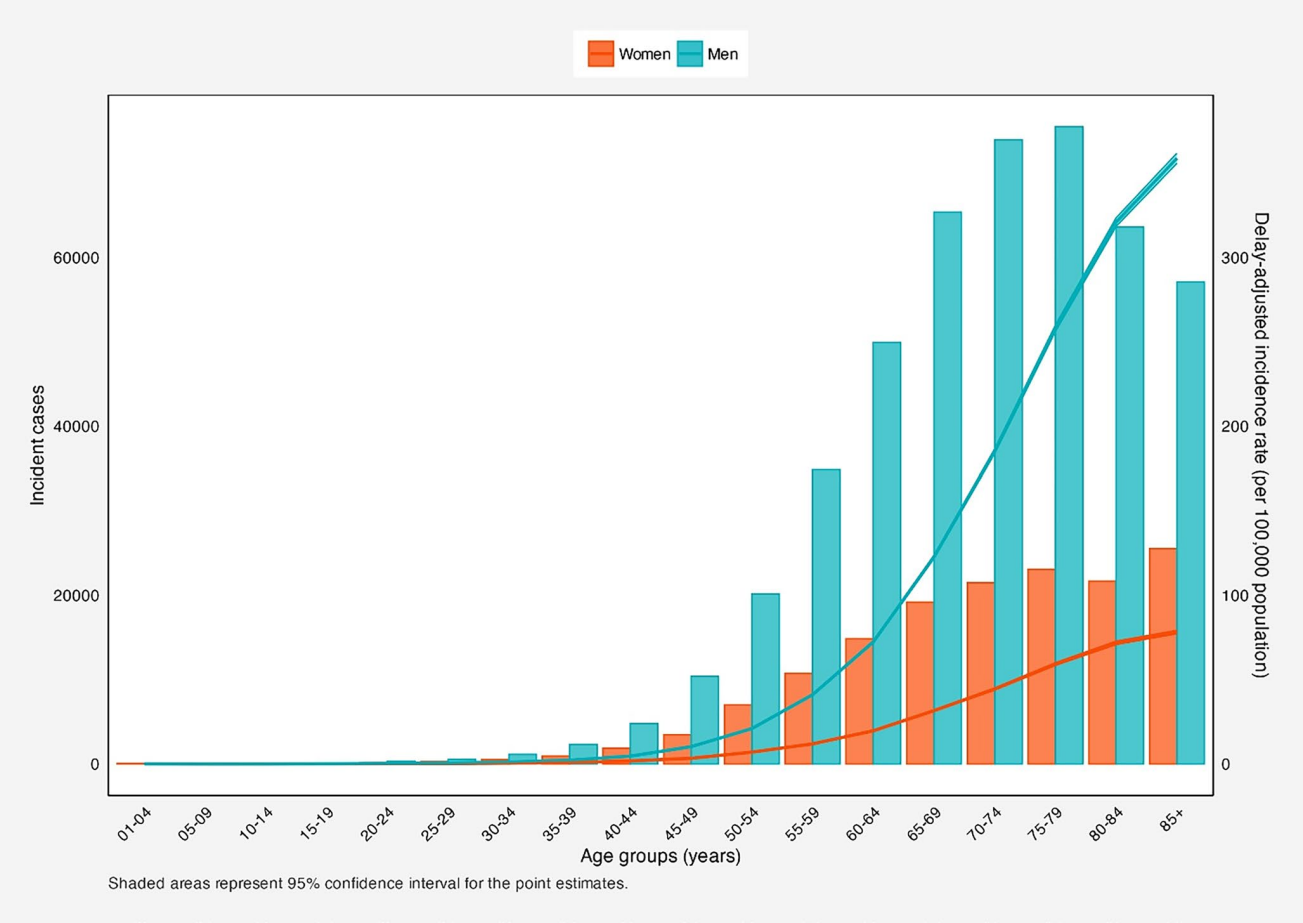
Incident numbers and delay‐adjusted incidence rate of bladder cancer in the United States among males and females in each age group. Shaded areas are the confidence interval range for the point estimates.

From 2000 to 2019, a total of 610 651 cases of bladder cancer were recorded in the US among all ages. The most commonly reported subtype was urothelial carcinoma (92.99%). Most cases were among Non‐Hispanic Whites (NHWs) (84.48%) and those aged 70 to 84 years (45.72%). Delayed ASIR per 100 000 population was 35.90 (35.79, 36.00) for men and 8.89 (8.85, 8.94) for women. NHW men had the highest ASIR (41.98 [41.85, 42.12]). The AAPC was −0.95% (−1.06, −0.83) for men and −1.02% (−1.21, −0.84) for women. Hispanic men had the greatest decrease in ASIR over 2000–2019 (AAPC: −1.20% [−1.41, −0.95]) (Table 1, Figure 1A,B). The overall parallel and identical trends of bladder cancer are provided in Table S1 and Table S2. Between 2000 and 2019, across all races and ethnicities, the delay‐adjusted incidence rate of bladder cancer increased with advancing age. Also, the incident cases peaked in 75–79 and > 85 age groups in males and females, respectively (Figure 2).

**TABLE 1:** Counts and age‐standardized rate of bladder cancer incidence per 100 000 and average annual percent change from 2000 to 2019 in the United States, by age, sex, and race.

Age group (years)	Men			Women		
	Case (%)	Delayed ASIR (95% CI)	AAPC (95% CI)	Case (%)	Delayed ASIR (95% CI)	AAPC (95% CI)
All race/ethnicities						
All	459 910 (75.31)	35.9 (35.79, 36)	−0.95 (−1.06, −0.83)	150 741 (24.69)	8.89 (8.85, 8.94)	−1.02 (−1.21, −0.84)
0 to 39	4496 (0.74)	0.57 (0.56, 0.59)	−1.39 (−2.06, −0.77)	1996 (0.33)	0.26 (0.24, 0.27)	−1.67 (−2.63, −0.52)
40 to 54	35 261 (5.774)	11.22 (11.1, 11.33)	−2.33 (−2.58, −2.1)	12 288 (2.01)	3.8 (3.73, 3.87)	−1.97 (−2.2, −1.76)
55 to 69	150 098 (24.58)	74.05 (73.67, 74.42)	−1.69 (−1.83, −1.55)	44 744 (7.33)	19.92 (19.74, 20.11)	−1.51 (−2.06, −1.01)
70 to 84	212 985 (34.88)	242.5 (241.47, 243.54)	−0.71 (−0.86, −0.56)	66 193 (10.84)	55.99 (55.56, 56.42)	−0.69 (−1.08, −0.36)
+85	57 070 (9.34)	358.73 (355.79, 361.69)	−0.04 (−0.4, 0.41)	25 520 (4.18)	78.15 (77.19, 79.11)	−0.59 (−0.8, −0.38)
Hispanic						
All	28 466 (73.56)	19.46 (19.22, 19.7)	−1.2 (−1.41, −0.95)	10 233 (26.44)	5.17 (5.07, 5.28)	−1.03 (−1.4, −0.62)
0 to 39	703 (1.82)	0.33 (0.3, 0.35)	0.2 (−1.15, 1.64)	372 (0.96)	0.18 (0.16, 0.2)	0.11 (−1.91, 2.42)
40 to 54	3128 (8.08)	5.33 (5.14, 5.52)	−2.53 (−3.26, −1.77)	1243 (3.21)	2.11 (2, 2.23)	−1.5 (−3.05, 0.17)
55 to 69	9730 (25.14)	37 (36.26, 37.75)	−1.49 (−2.06, −0.82)	3171 (8.19)	10.56 (10.19, 10.93)	−1.49 (−2.08, −0.81)
70 to 84	11 884 (30.71)	133.07 (130.67, 135.5)	−0.94 (−1.34, −0.49)	4062 (10.50)	32.44 (31.45, 33.46)	−0.73 (−1.31, −0.09)
+85	3021 (7.81)	220.98 (213.16, 229)	−1.26 (−1.79, −0.6)	1385 (3.58)	54.54 (51.7, 57.49)	−1.31 (−1.9, −0.63)
NHB						
All	21 731 (66.86)	20.17 (19.88, 20.45)	−0.19 (−0.66, 0.33)	10 770 (33.14)	6.77 (6.65, 6.91)	−1.02 (−1.43, −0.57)
0 to 39	303 (0.93)	0.34 (0.3, 0.38)	−0.98 (−3.25, 1.32)	143 (0.44)	0.14 (0.12, 0.17)	1.76 (−0.87, 4.76)
40 to 54	2538 (7.81)	7.41 (7.12, 7.7)	−1.69 (−2.59, −0.8)	1009 (3.10)	2.59 (2.43, 2.75)	−1.51 (−2.52, −0.51)
55 to 69	8664 (26.66)	45.6 (44.63, 46.57)	−0.25 (−0.71, 0.28)	3363 (10.35)	14.07 (13.6, 14.56)	−0.56 (−1.16, 0.12)
70 to 84	8401 (25.85)	128.5 (125.74, 131.3)	−0.07 (−0.72, 0.73)	4583 (14.10)	43.64 (42.38, 44.92)	−0.98 (−1.69, −0.23)
+85	1825 (5.62)	192.29 (183.56, 201.32)	0.71 (−0.43, 2.14)	1672 (5.14)	69.53 (66.23, 72.94)	−1.81 (−2.61, −0.97)
NHW						
All	391 847 (75.95)	41.98 (41.85, 42.12)	−0.66 (−0.79, −0.53)	124 080 (24.05)	10.34 (10.28, 10.39)	−0.69 (−0.89, −0.51)
0 to 39	3146 (0.61)	0.77 (0.75, 0.8)	−1.39 (−2.03, −0.8)	1361 (0.26)	0.34 (0.32, 0.36)	−1.68 (−2.98, −0.5)
40 to 54	27 741 (5.38)	14.26 (14.09, 14.43)	−1.73 (−2.09, −1.4)	9485 (1.84)	4.86 (4.76, 4.96)	−1.29 (−1.54, −1.05)
55 to 69	125 589 (24.34)	88.68 (88.19, 89.18)	−1.52 (−1.66, −1.37)	36 379 (7.05)	23.97 (23.73, 24.22)	−1.48 (−1.72, −1.23)
70 to 84	185 140 (35.88)	280 (278.73, 281.28)	−0.37 (−0.5, −0.25)	55 267 (10.71)	63.73 (63.2, 64.27)	−0.28 (−0.73, 0.08)
+85	50 231 (9.74)	401.35 (397.85, 404.88)	0.48 (0.1, 0.99)	21 588 (4.19)	83.4 (82.29, 84.52)	−0.1 (−0.33, 0.14)

Abbreviations: AAPC, Average annual percent change; ASIR, Age‐standardized incidence rate; CI, Confidence interval; NHB, Non‐Hispanic Black; NHW, Non‐Hispanic White.

There was a significant decrease in the ASIR of bladder cancer across all races/ethnicities in both sexes within all age groups (percent change [PC]: −6.23% [−7.69, −4.77]) and for males (PC: −5.58% [−7.27, −3.89]) and females (PC: −9.27% [−12.26, −6.29]) from 2019 to November 2020 (Table 2).

**TABLE 2:** Percent change in age‐standardized, delay‐adjusted incidence rates from 2019 to 2020 for all ages, by race, and sex, using the November 2022 data submission.

Races/ethnicities	Sex	2019 delayed ASIR (95% CI)	2020 delayed ASIR (95% CI)	PC (95% CI)
All	Both	18.46 (18.26, 18.66)	17.31 (17.11, 17.5)	−6.23 (−7.69, −4.77)
	Female	7.98 (7.81, 8.16)	7.24 (7.08, 7.41)	−9.27 (−12.26, −6.29)
	Male	31.92 (31.52, 32.32)	30.14 (29.75, 30.53)	−5.58 (−7.27, −3.89)
Hispanic	Both	10.4 (9.99, 10.82)	9.2 (8.82, 9.59)	−11.54 (−16.55, −6.53)
	Female	5.05 (4.67, 5.44)	4.18 (3.85, 4.54)	−17.23 (−26.22, −8.24)
	Male	17.49 (16.67, 18.34)	15.77 (15.01, 16.56)	−9.83 (−15.93, −3.74)
NHB	Both	11.48 (10.96, 12)	11.09 (10.58, 11.6)	−3.4 (−9.57, 2.77)
	Female	6.21 (5.73, 6.72)	5.81 (5.34, 6.3)	−6.44 (−17.02, 4.14)
	Male	19.44 (18.36, 20.56)	18.98 (17.92, 20.08)	−2.37 (−10.19, 5.45)
NHW	Both	22.39 (22.12, 22.67)	21.1 (20.83, 21.36)	−5.76 (−7.45, −4.08)
	Female	9.55 (9.31, 9.8)	8.68 (8.45, 8.92)	−9.11 (−12.44, −5.78)
	Male	38.36 (37.83, 38.9)	36.41 (35.89, 36.93)	−5.08 (−6.99, −3.18)

Abbreviations: ASIR, Age‐standardized incidence rate; CI, Confidence interval; NHB, Non‐Hispanic Black; NHW, Non‐Hispanic White; PC, percent change.

### 3.2 Subtype‐Specific Incidence Trends

#### 3.2.1 Declining Incidence of Urothelial Carcinoma, the Predominant Subtype With Highest Rates in Non‐Hispanic White Males

From 2000 to 2019, there were 567 841 urothelial carcinoma cases in all age groups in the US. The majority of cases were men (76.07%), NHWs (84.95%), and between 70 and 84 years (45.89%). The ASIR per 100 000 population was 33.54 (33.44, 33.64) for men and 8.01 (7.97, 8.05) for women. NHW men had the highest ASIR (39.36 [39.23, 39.49]). The AAPCs for men and women were −0.98% (−1.09, −0.86) and −0.99% (−1.16, −0.81), respectively, with Hispanic men having the greatest decrease compared to other groups (−1.32% [−1.62, −0.96]) (Table 3, Figures S1 and S2). The ASIR of urothelial carcinoma increased with advancing age and peaked in > 85 years in both males and females (Figure S3).

**TABLE 3:** Counts and age‐standardized rate of urothelial carcinoma incidence per 100 000 and average annual percent change from 2000 to 2019 in the United States, by age, sex, and race.

Age group (years)	Men			Women		
	Case (%)	ASIR (95% CI)	AAPC (95% CI)	Case (%)	ASIR (95% CI)	AAPC (95% CI)
All race/ethnicities						
All	431 937 (76.07)	33.54 (33.44, 33.64)	−0.98 (−1.09, −0.86)	135 904 (23.93)	8.01 (7.97, 8.05)	−0.99 (−1.16, −0.81)
0 to 39	4057 (0.71)	0.52 (0.5, 0.53)	−1.42 (−2, −0.85)	1655 (0.29)	0.21 (0.2, 0.22)	−1.78 (−2.98, −0.45)
40 to 54	33 263 (5.86)	10.55 (10.44, 10.67)	−2.38 (−2.68, −2.1)	10 983 (1.94)	3.38 (3.32, 3.45)	−2.09 (−2.45, −1.75)
55 to 69	142 695 (25.13)	70.19 (69.83, 70.56)	−1.62 (−1.84, −1.41)	41 508 (7.31)	18.43 (18.25, 18.61)	−1.41 (−1.92, −0.97)
70 to 84	200 618 (35.33)	227.7 (226.7, 228.7)	−0.7 (−0.91, −0.49)	60 457 (10.65)	51.03 (50.62, 51.44)	−0.61 (−0.97, −0.23)
+85	51 304 (9.03)	321.45 (318.68, 324.25)	−0.13 (−0.46, 0.26)	21 301 (3.75)	65.04 (64.17, 65.92)	−0.65 (−0.95, −0.32)
Hispanic						
All	26 157 (74.83)	17.73 (17.5, 17.96)	−1.32 (−1.62, −0.96)	8797 (25.17)	4.42 (4.32, 4.51)	−1.18 (−1.57, −0.74)
0 to 39	594 (1.70)	0.28 (0.26, 0.3)	−0.1 (−1.95, 1.95)	287 (0.82)	0.14 (0.12, 0.16)	0.35 (−2.81, 3.94)
40 to 54	2869 (8.21)	4.86 (4.69, 5.04)	−2.72 (−3.36, −2.04)	1022 (2.923)	1.73 (1.62, 1.84)	−2 (−3.65, −0.22)
55 to 69	9018 (25.80)	34.07 (33.36, 34.78)	−1.58 (−2.2, −0.86)	2853 (8.162)	9.43 (9.09, 9.78)	−1.55 (−2.23, −0.77)
70 to 84	11 037 (31.58)	122.76 (120.47, 125.09)	−1 (−1.46, −0.47)	3551 (10.16)	28.17 (27.25, 29.11)	−0.79 (−1.47, −0.01)
+85	2639 (7.55)	191.69 (184.45, 199.15)	−1.58 (−2.25, −0.72)	1084 (3.10)	42.38 (39.89, 44.98)	−1.74 (−2.54, −0.77)
NHB						
All	19 548 (68.40)	17.95 (17.68, 18.22)	−0.31 (−0.85, 0.23)	9035 (31.60)	5.66 (5.55, 5.78)	−0.89 (−1.38, −0.35)
0 to 39	237 (0.83)	0.27 (0.23, 0.3)	−5.16 (−10.13, −1.86)	97 (0.34)	0.1 (0.08, 0.12)	2.53 (−0.33, 6.07)
40 to 54	2264 (7.92)	6.58 (6.31, 6.86)	−1.83 (−2.91, −0.77)	777 (2.72)	1.98 (1.84, 2.12)	−1.45 (−2.79, −0.09)
55 to 69	7929 (27.74)	41.48 (40.57, 42.41)	−0.26 (−0.72, 0.26)	2882 (10.08)	12.01 (11.57, 12.46)	−0.4 (−0.96, 0.26)
70 to 84	7567 (26.473)	114.99 (112.39, 117.64)	−0.01 (−0.66, 0.71)	3950 (13.82)	37.44 (36.28, 38.62)	−0.77 (−1.52, 0.04)
+85	1551 (5.43)	162.46 (154.47, 170.75)	0.66 (−0.26, 1.88)	1329 (4.65)	55 (52.09, 58.04)	−2.05 (−3.01, −1.05)
NHW						
All	369 454 (76.59)	39.36 (39.23, 39.49)	−0.71 (−0.81, −0.61)	112 956 (23.41)	9.41 (9.35, 9.47)	−0.67 (−0.9, −0.38)
0 to 39	2919 (0.61)	0.72 (0.69, 0.74)	−1.37 (−2.27, −0.55)	1173 (0.24)	0.29 (0.28, 0.31)	−1.92 (−3.64, −0.26)
40 to 54	26 391 (5.47)	13.52 (13.35, 13.68)	−1.75 (−2.12, −1.4)	8700 (1.80)	4.44 (4.34, 4.53)	−1.38 (−1.71, −1.06)
55 to 69	119 930 (24.86)	84.35 (83.87, 84.83)	−1.49 (−1.67, −1.34)	34 080 (7.06)	22.37 (22.13, 22.61)	−1.27 (−1.99, −0.7)
70 to 84	174 900 (36.26)	263.42 (262.18, 264.66)	−0.4 (−0.58, −0.22)	50 867 (10.54)	58.49 (57.98, 59.01)	−0.23 (−0.91, 0.34)
+85	45 314 (9.40)	360.41 (357.1, 363.74)	0.21 (−0.2, 0.71)	18 136 (3.76)	69.78 (68.77, 70.8)	−0.13 (−0.41, 0.16)

Abbreviations: AAPC, Average annual percent change; ASIR, Age‐standardized incidence rate; CI, Confidence interval; N/A, Not available; NHB, Non‐Hispanic Black; NHW, Non‐Hispanic White.

#### 3.2.2 Decreasing Incidence of Squamous Cell Carcinoma, More Prevalent in Women and Non‐Hispanic Blacks With Greatest Declines in Non‐Hispanic Black Males

Over 2000–2019, there were a total of 5415 cases of SCC in the US across all age groups. The majority of cases were in women (51.76%), NHWs (76.19%), and individuals aged 70–84 years (43.32%). The ASIR per 100 000 population was 0.21 (0.20, 0.21) for men and 0.16 (0.16, 0.17) for women. The AAPC for men and women were −2.59% (−3.53, −1.65) and −1.77% (−2.60, −0.94), respectively. Non‐Hispanic Black (NHB) men had the highest ASIR (0.26 [0.23, 0.30]) and experienced the greatest significant decline over 2000–2019 (AAPC: −3.80 [−6.24, −1.38]) (Table 4 and Figure S4).

**TABLE 4:** Counts and age‐standardized rate of squamous cell carcinoma incidence per 100 000 and average annual percent change from 2000 to 2019 in the United States, by age, sex, and race.

Age group (years)	Men			Women		
	Case (%)	ASIR (95% CI)	AAPC (95% CI)	Case (%)	ASIR (95% CI)	AAPC (95% CI)
All race/ethnicities						
All	2612 (48.24)	0.21 (0.2, 0.21)	−2.59 (−3.53, −1.65)	2803 (51.76)	0.16 (0.16, 0.17)	−1.77 (−2.6, −0.94)
0 to 39	30 (0.56)	0 (0, 0.01)	N/A	56 (1.03)	0.01 (0.01, 0.01)	N/A
40 to 54	227 (4.192)	0.07 (0.06, 0.08)	−4.33 (−10.34, 1.29)	309 (5.71)	0.1 (0.09, 0.11)	−1.16 (−3.49, 1.17)
55 to 69	751 (13.87)	0.37 (0.34, 0.39)	−2.08 (−3.71, −0.31)	728 (13.44)	0.32 (0.3, 0.35)	−1.79 (−3.43, 0.01)
70 to 84	1206 (22.27)	1.37 (1.3, 1.45)	−2.84 (−4.53, −1.18)	1140 (21.05)	0.96 (0.9, 1.01)	−1.37 (−2.99, 0.26)
+85	398 (7.35)	2.49 (2.25, 2.75)	−2.6 (−4.61, −0.44)	570 (10.53)	1.74 (1.6, 1.89)	−2.81 (−5.06, −0.55)
Hispanic						
All	211 (43.42)	0.14 (0.12, 0.16)	−1.45 (−3.29, 0.76)	275 (56.58)	0.14 (0.12, 0.16)	−1.63 (−4.06, 1.23)
0 to 39	3 (0.62)	0 (0, 0)	N/A	14 (2.88)	0.01 (0, 0.01)	N/A
40 to 54	26 (5.35)	0.04 (0.03, 0.06)	N/A	37 (7.61)	0.06 (0.04, 0.09)	N/A
55 to 69	74 (15.23)	0.28 (0.22, 0.35)	N/A	75 (15.43)	0.25 (0.2, 0.32)	−2.56 (−6.25, 1.84)
70 to 84	85 (17.49)	0.94 (0.75, 1.16)	−2.11 (−5.79, 2.16)	113 (23.25)	0.9 (0.74, 1.09)	−0.76 (−3.92, 3.14)
+85	23 (4.73)	1.67 (1.06, 2.51)	N/A	36 (7.41)	1.41 (0.99, 1.95)	N/A
NHB						
All	272 (42.43)	0.26 (0.23, 0.3)	−3.8 (−6.24, −1.38)	369 (57.57)	0.23 (0.2, 0.25)	−2.74 (−4.82, −0.59)
0 to 39	10 (1.56)	0.01 (0.01, 0.02)	N/A	7 (1.09)	0.01 (0, 0.01)	N/A
40 to 54	34 (5.30)	0.1 (0.07, 0.14)	N/A	55 (8.58)	0.14 (0.11, 0.18)	N/A
55 to 69	85 (13.26)	0.45 (0.36, 0.56)	N/A	114 (17.79)	0.47 (0.38, 0.56)	−2.83 (−5.89, 0.59)
70 to 84	117 (18.25)	1.78 (1.47, 2.14)	−3.35 (−6.91, 0.25)	143 (22.31)	1.36 (1.15, 1.6)	−1.66 (−5.56, 2.67)
+85	26 (4.06)	2.72 (1.78, 3.99)	N/A	50 (7.80)	2.07 (1.54, 2.73)	N/A
NHW						
All	2034 (49.30)	0.22 (0.21, 0.23)	−2.3 (−3.39, −1.22)	2092 (50.70)	0.17 (0.17, 0.18)	−1.38 (−2.66, −0.21)
0 to 39	15 (0.36)	0 (0, 0.01)	N/A	31 (0.75)	0.01 (0.01, 0.01)	N/A
40 to 54	162 (3.93)	0.08 (0.07, 0.1)	−1.96 (−6.33, 1.96)	211 (5.11)	0.11 (0.1, 0.13)	−0.58 (−4.42, 3.06)
55 to 69	567 (13.74)	0.4 (0.36, 0.43)	−1.56 (−3.33, 0.35)	521 (12.63)	0.34 (0.31, 0.37)	−1.56 (−3.41, 0.46)
70 to 84	958 (23.22)	1.45 (1.36, 1.54)	−2.64 (−4.65, −0.74)	852 (20.65)	0.97 (0.9, 1.03)	−1.08 (−3.09, 0.94)
+85	332 (8.05)	2.64 (2.36, 2.94)	−2.41 (−4.64, −0.08)	477 (11.56)	1.84 (1.67, 2.01)	−2.05 (−4.44, 0.31)

Abbreviations: AAPC, Average annual percent change; ASIR, Age‐standardized incidence rate; CI, Confidence interval; N/A, Not available; NHB, Non‐Hispanic Black; NHW, Non‐Hispanic White.

The ASIR of SCC increased with advancing age, while there were not significant differences between males and females. Also, the highest number of incident cases was among 75–79‐year‐old males and > 85‐year‐old females (Figure S5).

#### 3.2.3 Decreasing Incidence of Adenocarcinoma, With Higher Rates in Non‐Hispanic Black Males and Greatest Declines in Non‐Hispanic Black Females

Over 2000–2019, 4487 cases of adenocarcinoma in all age groups in the US were reported. The majority of the cases were men (62.56%), NHWs (68.39%), and aged between 70 and 84 years (36.02%). The ASIR per 100 000 population was 0.21 (0.21, 0.22) for men and 0.10 (0.10, 0.11) for women. NHB men had the highest ASIR (0.29 [0.25, 0.32]). The ASIR decreased in both sexes over 2000–2019 (AAPC: −1.29% [−2.45, −0.06] and AAPC: −1.90% [−2.88, −0.29] for men and women, respectively). NHB women experienced the greatest decrease among other groups between 2000 and 2019 (AAPC: −2.70% [−4.49, −0.87]) (Table 5, Figures S6 and S7).

**TABLE 5:** Counts and age‐standardized rate of adenocarcinoma incidence per 100 000 and average annual percent change from 2000 to 2019 in the United States, by age, sex, and race.

Age group (years)	Men			Women		
	Case (%)	ASIR (95% CI)	AAPC (95% CI)	Case (%)	ASIR (95% CI)	AAPC (95% CI)
All race/ethnicities						
All	2807 (62.56)	0.21 (0.21, 0.22)	−1.29 (−2.45, −0.06)	1680 (37.44)	0.1 (0.1, 0.11)	−1.9 (−2.88, −0.29)
0 to 39	110 (2.45)	0.01 (0.01, 0.02)	2.17 (−0.44, 5.17)	75 (1.67)	0.01 (0.01, 0.01)	2.8 (−3.68, 7.35)
40 to 54	429 (9.56)	0.14 (0.13, 0.15)	−1.61 (−3.66, 0.33)	288 (6.42)	0.09 (0.08, 0.1)	1.33 (−1.45, 4.37)
55 to 69	903 (20.12)	0.44 (0.41, 0.47)	−0.69 (−2.31, 1.17)	541 (12.06)	0.24 (0.22, 0.26)	−0.11 (−2.02, 2.17)
70 to 84	1057 (23.56)	1.2 (1.13, 1.27)	−2.5 (−3.88, −0.5)	559 (12.46)	0.47 (0.43, 0.51)	−2.96 (−4.45, −1.35)
+85	308 (6.86)	1.93 (1.72, 2.16)	−1.84 (−4.74, 1.42)	217 (4.84)	0.66 (0.58, 0.76)	−2.97 (−5.97, −0.08)
Hispanic						
All	316 (60.54)	0.18 (0.16, 0.2)	−2.66 (−5.6, 0.89)	206 (39.46)	0.09 (0.08, 0.1)	−0.39 (−3.52, 2.36)
0 to 39	37 (7.09)	0.02 (0.01, 0.02)	N/A	26 (4.98)	0.01 (0.01, 0.02)	N/A
40 to 54	75 (14.37)	0.13 (0.1, 0.16)	0.73 (−3.57, 6.08)	65 (12.45)	0.11 (0.08, 0.14)	N/A
55 to 69	88 (16.86)	0.33 (0.26, 0.41)	−2.54 (−7.63, 3.92)	52 (9.96)	0.17 (0.13, 0.22)	N/A
70 to 84	93 (17.816)	1 (0.8, 1.22)	−3.25 (−6.21, 0)	50 (9.58)	0.39 (0.29, 0.52)	N/A
+85	23 (4.406)	1.67 (1.06, 2.51)	N/A	13 (2.49)	0.51 (0.27, 0.87)	N/A
NHB						
All	345 (54.00)	0.29 (0.25, 0.32)	−1.87 (−3.56, −0.02)	294 (46.00)	0.17 (0.15, 0.19)	−2.7 (−4.49, −0.87)
0 to 39	12 (1.88)	0.01 (0.01, 0.02)	N/A	12 (1.88)	0.01 (0.01, 0.02)	N/A
40 to 54	71 (11.11)	0.21 (0.16, 0.26)	N/A	58 (9.08)	0.15 (0.11, 0.2)	N/A
55 to 69	143 (22.38)	0.74 (0.62, 0.87)	−1.17 (−5.71, 4.24)	117 (18.31)	0.48 (0.4, 0.58)	−0.72 (−3.09, 2.13)
70 to 84	104 (16.27)	1.56 (1.27, 1.89)	−1.74 (−4.73, 1.75)	84 (13.14)	0.79 (0.63, 0.98)	−3.76 (−7, −0.76)
+85	15 (2.35)	1.57 (0.88, 2.59)	N/A	23 (3.60)	0.95 (0.6, 1.43)	N/A
NHW						
All	1984 (64.64)	0.21 (0.2, 0.22)	−1.23 (−2.69, 0.3)	1085 (35.36)	0.09 (0.09, 0.1)	−2.16 (−3.43, 0.01)
0 to 39	49 (1.60)	0.01 (0.01, 0.02)	N/A	26 (0.85)	0.01 (0, 0.01)	N/A
40 to 54	253 (8.24)	0.13 (0.12, 0.15)	−3.08 (−6.33, −0.35)	146 (4.76)	0.08 (0.06, 0.09)	2.8 (−0.92, 6.91)
55 to 69	616 (20.07)	0.43 (0.4, 0.47)	−0.53 (−2.59, 1.86)	339 (11.05)	0.22 (0.2, 0.25)	−2.6 (−4.42, 1.09)
70 to 84	810 (26.39)	1.22 (1.14, 1.31)	−1.35 (−3.14, 0.52)	402 (13.10)	0.46 (0.42, 0.51)	−2.87 (−4.98, −0.32)
+85	256 (8.34)	2.04 (1.79, 2.3)	−0.85 (−3.94, 2.74)	172 (5.60)	0.66 (0.57, 0.77)	−2.99 (−6.49, 0.21)

Abbreviations: AAPC, Average annual percent change; ASIR, Age‐standardized incidence rate; CI, Confidence interval; N/A, Not available; NHB, Non‐Hispanic Black; NHW, Non‐Hispanic White.

The incident cases and rates were higher among women than men. Also, the highest ASIR for both men and women was reported in those > 85 years (Figure S8).

#### 3.2.4 Increasing Incidence of Small Cell Neuroendocrine Carcinoma, Predominantly in Males With Highest Rates in Non‐Hispanic Whites

From 2000 to 2019, there were 3588 small cell neuroendocrine carcinoma cases in all age groups in the US. The majority of cases were men (77.73%), NHWs (83.89%), and between 70 and 84 years (47.43%). The ASIR per 100 000 population was 0.22 (0.21, 0.23) for men and 0.05 (0.04, 0.05) for women. NHW men had the highest ASIR (0.26 [0.25, 0.27]). AAPC for men was 4.86% (3.24, 7.52) and it was 3.73% (2.24, 5.58) for women (Table 6 and Figure S9).

**TABLE 6:** Counts and age‐standardized rate of small cell neuroendocrine carcinoma incidence per 100 000 and average annual percent change from 2000 to 2019 in the United States, by age, sex, and race.

Age group (years)	Men			Women		
	Case (%)	ASIR (95% CI)	AAPC (95% CI)	Case (%)	ASIR (95% CI)	AAPC (95% CI)
All race/ethnicities						
All	2789 (77.73)	0.22 (0.21, 0.23)	4.86 (3.24, 7.52)	799 (22.27)	0.05 (0.04, 0.05)	3.73 (2.24, 5.58)
0 to 39	10 (0.28)	0 (0, 0)	N/A	7 (0.20)	0 (0, 0)	N/A
40 to 54	173 (4.82)	0.05 (0.05, 0.06)	1.9 (−2.12, 6.55)	42 (1.17)	0.01 (0.01, 0.02)	N/A
55 to 69	823 (22.94)	0.41 (0.38, 0.43)	3.28 (1.8, 5.26)	218 (6.08)	0.1 (0.08, 0.11)	2.58 (−0.06, 6.21)
70 to 84	1323 (36.87)	1.5 (1.42, 1.59)	4.6 (3.34, 6.45)	379 (10.56)	0.32 (0.29, 0.35)	4.65 (2.97, 6.83)
+85	460 (12.82)	2.88 (2.62, 3.16)	5.46 (2.94, 9.3)	153 (4.26)	0.47 (0.4, 0.55)	N/A
Hispanic						
All	184 (69.43)	0.13 (0.11, 0.15)	N/A	81 (30.57)	0.04 (0.03, 0.05)	N/A
0 to 39	1 (0.38)	0 (0, 0)	N/A	3 (1.13)	0 (0, 0)	N/A
40 to 54	19 (7.17)	0.03 (0.02, 0.05)	N/A	6 (2.27)	0.01 (0, 0.02)	N/A
55 to 69	69 (26.04)	0.26 (0.2, 0.33)	N/A	25 (9.43)	0.08 (0.05, 0.12)	N/A
70 to 84	74 (27.92)	0.83 (0.65, 1.05)	N/A	34 (12.83)	0.27 (0.19, 0.38)	N/A
+85	21 (7.92)	1.53 (0.94, 2.33)	N/A	13 (4.91)	0.51 (0.27, 0.87)	N/A
NHB						
All	132 (67.35)	0.13 (0.11, 0.16)	5.63 (1.91, 11.42)	64 (32.65)	0.04 (0.03, 0.05)	N/A
0 to 39	0 (0)	0 (0, 0)	N/A	0 (0)	0 (0, 0)	N/A
40 to 54	11 (5.61)	0.03 (0.02, 0.06)	N/A	5 (2.55)	0.01 (0, 0.03)	N/A
55 to 69	48 (24.49)	0.26 (0.19, 0.34)	N/A	14 (7.14)	0.06 (0.03, 0.1)	N/A
70 to 84	55 (28.06)	0.82 (0.62, 1.07)	N/A	37 (18.88)	0.35 (0.24, 0.48)	N/A
+85	18 (9.19)	1.89 (1.12, 2.98)	N/A	8 (4.08)	0.33 (0.14, 0.65)	N/A
NHW						
All	2377 (78.97)	0.26 (0.25, 0.27)	4.88 (3.71, 6.8)	633 (21.03)	0.05 (0.05, 0.06)	3.92 (2.15, 6.09)
0 to 39	9 (0.3)	0 (0, 0)	N/A	4 (0.13)	0 (0, 0)	N/A
40 to 54	134 (4.45)	0.07 (0.06, 0.08)	1.98 (−2.01, 6.3)	30 (1.00)	0.02 (0.01, 0.02)	N/A
55 to 69	672 (22.32)	0.47 (0.44, 0.51)	3.53 (2.06, 5.43)	174 (5.78)	0.11 (0.1, 0.13)	2.79 (−0.3, 7)
70 to 84	1147 (38.11)	1.73 (1.63, 1.83)	4.49 (2.42, 7.8)	299 (9.93)	0.34 (0.31, 0.38)	4.82 (3.18, 6.86)
+85	415 (13.79)	3.3 (2.99, 3.63)	6.09 (3.81, 9.55)	126 (4.19)	0.48 (0.4, 0.58)	N/A

Abbreviations: AAPC, Average annual percent change; ASIR, Age‐standardized incidence rate; CI, Confidence interval; N/A, Not available; NHB, Non‐Hispanic Black; NHW, Non‐Hispanic White.

The incident cases and rates of bladder small cell neuroendocrine carcinoma were substantially higher among males, and it generally increased with aging (Figure S10).

#### 3.2.5 Stable to Slightly Decreasing Incidence of Sarcoma, Rare Subtype With Bimodal Age Peaks and Highest Rates in Non‐Hispanic Black Males

Over 2000–2019, there were a total of 592 cases of bladder sarcoma in the US across all age groups. The majority of cases were observed in men (61.65%), NHWs (66.72%), and cases between 70 and 84 years (28.03%). ASIR per 100 000 population was 0.03 (0.02, 0.03) for men and 0.01 (0.01, 0.02) for women. The AAPCs for men and women were −1.00% (−3.34, 1.50) and −2.03% (−4.54, 0.43), respectively. NHB men had the highest ASIR (0.04 [0.03, 0.05]) (Table 7 and Figure S11).

**TABLE 7:** Counts and age‐standardized rate of sarcoma incidence per 100 000 and average annual percent change from 2000 to 2019 in the United States, by age, sex, and race.

Age group (years)	Men			Women		
	Case (%)	ASIR (95% CI)	AAPC (95% CI)	Case (%)	ASIR (95% CI)	AAPC (95% CI)
All race/ethnicities						
All	365 (61.65)	0.03 (0.02, 0.03)	−1 (−3.34, 1.5)	227 (38.35)	0.01 (0.01, 0.02)	−2.03 (−4.54, 0.43)
0 to 39	101 (17.06)	0.01 (0.01, 0.01)	1.54 (−2.9, 6.81)	63 (10.64)	0.01 (0.01, 0.01)	N/A
40 to 54	41 (6.925)	0.01 (0.01, 0.02)	N/A	39 (6.59)	0.01 (0.01, 0.02)	N/A
55 to 69	80 (13.51)	0.04 (0.03, 0.05)	−1.72 (−4.48, 1.34)	34 (5.74)	0.02 (0.01, 0.02)	N/A
70 to 84	105 (17.736)	0.12 (0.1, 0.15)	−2.64 (−5.89, 0.56)	61 (10.30)	0.05 (0.04, 0.07)	−1.81 (−6.81, 3.08)
+85	38 (6.42)	0.24 (0.17, 0.33)	N/A	30 (5.07)	0.09 (0.06, 0.13)	N/A
Hispanic						
All	60 (59.40)	0.03 (0.02, 0.04)	N/A	41 (40.60)	0.02 (0.01, 0.02)	N/A
0 to 39	26 (25.75)	0.01 (0.01, 0.01)	N/A	18 (17.82)	0.01 (0, 0.01)	N/A
40 to 54	5 (4.95)	0.01 (0, 0.02)	N/A	7 (6.93)	0.01 (0, 0.02)	N/A
55 to 69	12 (11.88)	0.05 (0.02, 0.08)	N/A	5 (4.95)	0.02 (0.01, 0.04)	N/A
70 to 84	13 (12.87)	0.15 (0.08, 0.26)	N/A	10 (9.90)	0.08 (0.04, 0.15)	N/A
+85	4 (3.96)	0.29 (0.08, 0.74)	N/A	1 (0.99)	0.04 (0, 0.22)	N/A
NHB						
All	45 (61.64)	0.04 (0.03, 0.05)	N/A	28 (38.36)	0.02 (0.01, 0.02)	N/A
0 to 39	17 (23.28)	0.02 (0.01, 0.03)	N/A	9 (12.33)	0.01 (0, 0.02)	N/A
40 to 54	2 (2.74)	0.01 (0, 0.02)	N/A	7 (9.59)	0.02 (0.01, 0.04)	N/A
55 to 69	12 (16.44)	0.06 (0.03, 0.11)	N/A	4 (5.48)	0.02 (0, 0.04)	N/A
70 to 84	8 (10.96)	0.12 (0.05, 0.24)	N/A	6 (8.22)	0.06 (0.02, 0.13)	N/A
+85	6 (8.22)	0.63 (0.23, 1.37)	N/A	2 (2.74)	0.08 (0.01, 0.3)	N/A
NHW						
All	243 (61.52)	0.03 (0.02, 0.03)	−1.48 (−3.78, 0.71)	152 (38.48)	0.02 (0.01, 0.02)	−2.88 (−6.12, −0.01)
0 to 39	49 (12.41)	0.01 (0.01, 0.02)	N/A	34 (8.61)	0.01 (0.01, 0.01)	N/A
40 to 54	30 (7.594)	0.02 (0.01, 0.02)	N/A	22 (5.57)	0.01 (0.01, 0.02)	N/A
55 to 69	55 (13.92)	0.04 (0.03, 0.05)	−2.48 (−7.08, 2.57)	25 (6.33)	0.02 (0.01, 0.02)	N/A
70 to 84	81 (20.51)	0.12 (0.1, 0.15)	−1.45 (−4.99, 2.18)	45 (11.39)	0.05 (0.04, 0.07)	−4.05 (−8.8, −0.12)
+85	28 (7.09)	0.22 (0.15, 0.32)	N/A	26 (6.58)	0.1 (0.07, 0.15)	N/A

Abbreviations: AAPC, Average annual percent change; ASIR, Age‐standardized incidence rate; CI, Confidence interval; N/A, Not available; NHB, Non‐Hispanic Black; NHW, Non‐Hispanic White.

The highest incident cases of bladder sarcoma were reported in 1–4 and > 85 years for men and women, respectively. Also, the highest incidence rate was in 85+ age groups for both males and females (Figure S12).

## 4 Discussion

### 4.1 Overall Incidence Trends and Comparison With Previous Studies

Between 2000 and 2019, the US witnessed a substantial burden of bladder cancer, with over 610 000 cases reported, predominantly as urothelial carcinoma. The incidence of bladder cancer was significantly higher among NHWs and in the older population, especially those aged 70 to 84 years. Notably, men were more affected than women, constituting 75.31% of bladder cancer cases. Also, there was a significant decrease in ASIR of bladder cancer over 2000–2019 and during the COVID‐19 pandemic in the US.

Results of the global burden of diseases project reported the incident cases and the ASIR of bladder cancer in the US as 49 737 cases and 8.8 per 100 000, respectively [30]. However, our study based on the reported data of the SEER revealed more than 610 thousand cases of bladder cancer over 2000–2019 in the US. Additionally, the study by Safiri et al. showed a non‐significant 4.3% increase in ASIR of bladder cancer between 1990 and 2019 in the US, while we found a significant decrease in ASIR of bladder cancer in both males and females. There are some explanations for the differences in observations between the studies. First, our study used the real data, while the abovementioned study developed modelling statistics to estimate the incidence [30]. Second, the AAPCs were reported for different time periods (1990–2019 vs. 2000–2019) [30]. Schafer and colleagues reported that the incidence rate of bladder cancer among males and females was 32.9 and 8.20 per 100 000 over 2015–2019 in the US, respectively [8]. In accordance with the findings, our results based on data over 2000–2019 showed almost similar values for males (35.90 per 100 000) and females (8.89 per 100 000) [8]. A previous report of SEER showed more than 222 thousand cases of bladder cancer over 2004–2016 [31], while we found above 610 thousand cases over 2000–2019. Although there are differences in the time frames between the studies, it seems that the incident cases of bladder cancer have increased. Higher exposure to risk factors, especially different forms of smoking, during the recent years partially explain the increase in the numbers of bladder cancers [32]. Therefore, targeted screening for high‐risk individuals based on their occupational risk factors and history of smoking may be considered for early detection and treatment [33].

### 4.2 Sex Disparity and the Role of Smoking

Over 2000–2019, we found that ASIR of bladder cancer among all races in all ages was about four times higher in males than females. Over 2015–2019, there was also a similar male to female ratio of 4.04 for bladder cancer among all races and ages in the US [8]. This sex disparity in bladder cancer incidence can be attributed primarily to varied rates of tobacco smoking, a known risk factor for bladder cancer [34]. In the US, tobacco smoke is estimated to be responsible for 50% to 65% of bladder cancer cases, marking it as the most significant risk factor for this disease [35]. During the 2000–2020 period, several tobacco control policies were implemented, including the 2009 Family Smoking Prevention and Tobacco Control Act, which mandated graphic health warnings, banned flavored cigarettes except menthol, restricted youth‐targeted marketing, and increased federal cigarette taxes, expanded smoke‐free air laws, state‐level excise tax increases, and funded cessation programs. These measures contributed to a decline in current use of tobacco products from about 32% to about 23% from 2000 to 2020 [36]. This smoking decline might partly play a role in the downward trends in bladder cancer ASIR over 2000–2019. However, contemporary data suggest that the risk attributed to smoking is now comparable between men and women and remains a substantial burden driving bladder cancer incidence [37]. Even further, one study showed that nearly 40% of bladder cancer cases in the US can be attributed to smoking [38]. Although men have a higher incidence of bladder cancer, women often have worse survival and clinical outcomes [39]. In our study, women indeed had lower overall incidence rates and demonstrated significant decreases, particularly among NHBs and Hispanics. It is important to understand that despite their lower incidence, women are more likely to be diagnosed with MIBC [39]. Regionalization and centralization of care for bladder cancer as well as improved medical education with greater enrollment of women in clinical bladder cancer trials may improve outcomes, reducing the gaps in sex disparities.

### 4.3 Age and Race/Ethnicity Disparities

Our analysis further revealed that age plays a role in cancer incidence, with the highest ASIR observed in individuals over 85 years across all subtypes of bladder cancer types and among both sexes. In this regard, a previous study reporting the global incidence rates of bladder cancer in 2019 showed that the highest incidence rate among both males and females was in those > 95 years [40]. Also, the highest incident cases were among 70–74 and 75–79 age groups for males and females, respectively [40]. There was also a similar pattern of incidence rates in favor of the elderly for other types of genitourinary cancers like prostate and kidney cancers globally and in the US [40, 41]. Despite the overall decline in incidence rates, the data suggest a growing cancer burden among the oldest age groups, which could reflect the aging population and potential delayed effects of exposure to risk factors. Understanding demographic factors must be integral to developing public health strategies and policies to address these disparities and changes in bladder cancer incidence rates.

The impact of race/ethnicity was also evident, with NHW men experiencing the highest ASIR. This aligns with Javier‐DesLoges et al. who demonstrated that NHWs were the highest race to be diagnosed with bladder cancer [42]. In accordance with our findings that showed that 84.48% of total cases of bladder cancer among NHWs, a previous study using SEER data between 1973 and 2014 reported that NHWs were the most common male patients (75.5%) [43]. Notably, when stratifying by race, smoking has contributed to the highest proportion of bladder cancer cases among American Indian/Alaska Natives (43%) and Whites (36%) for females, and was highest among American Indian/Alaska Natives (47%) and Blacks (44%) for males [38]. These impacts that smoking has on the incidence of bladder cancer greatly highlight the disparities present in the US. However, despite the high incidence in our study, there was a general decline in the ASIR across most demographics, with Hispanic men showing the greatest reduction. This downward trend reflects potential improvements in either risk‐factor modification, early cancer detection, or both. This prompts further investigation into the progress that the field of urology and oncology has had in reducing bladder cancer incidence. However, the race disparity's definite reason has not yet been identified and needs further investigations [43].

### 4.4 Impact of the COVID‐19 Pandemic on Incidence

The COVID‐19 pandemic has influenced the reporting and diagnosis of bladder cancer, with a notable decrease in ASIR across all races, ethnicities, and sexes. In this regard, a SEER‐based study reported an 8.02% decrease in ASIR of myeloma from 2019 to 2020 for both sexes combined in the US [44]. We also found a 6.23% decrease for bladder cancer in the US over the COVID‐19 pandemic. This might be attributed to the pandemic's impact on global healthcare systems, leading to delayed or missed cancer diagnoses [45], as evidenced by widespread reductions in screenings and early‐stage detections across multiple cancer types during 2020 [46, 47]. Follow‐up data showed partial recovery in overall cancer incidence by 2021 (nonsignificant −0.2% vs. projections), though deficits persisted for certain sites and demographics, highlighting the need for ongoing monitoring [48].

### 4.5 Histological Subtype Trends

With variant histology comprising nearly 25% of bladder cancer tumors, there is a paucity of literature regarding incidence rates and specific temporal, demographic patterns in histological subtypes of bladder cancer [49]. When considering the different histological subtypes of bladder cancer in our study, urothelial carcinoma remained the most reported (92.99%). In the same way with our results, the article by Al‐Husseini et al. showed that the highest ASIR of transitional cell carcinoma or urothelial carcinoma was among males (47.21 per 100 000), Caucasians (28.77 per 100 000), and > 84 years (134.61 per 100 000) over 1973–2014 in the US [50]. Another study using the California cancer registry showed the highest number of bladder cancer cases were urothelial carcinomas, followed by SCC and adenocarcinoma over 1988–2012 [51]. Although less prevalent, SCC significantly declined incidence rates, especially among men. SCC has been shown to have significantly higher incidence rates in black patients, particularly with higher rates of endemic schistosomiasis [43]. However, in the US and other western countries, non‐schistosomiasis SCC is often associated with spinal cord injury requiring chronic indwelling catheters [52]. In contrast, our study found that adenocarcinoma presented a more stable pattern with minor decreases in ASIR over time. Small cell neuroendocrine carcinoma, albeit rarer, followed the general trend of higher incidence in men and NHWs. Also, sarcoma is another rare subtype of bladder cancer which was not mostly evaluated in previous studies. However, there is a need to report and plan for the needs of patients with these rare forms of bladder cancer.

### 4.6 Strengths and Limitations

There are some limitations to this study. First, we did not perform analysis by different stages of bladder cancer in the US. As the study focused on incidence trends of bladder cancer, the incidence rates attributable to each risk factor were not provided. Second, there are several reporting sources for the SEER data such as laboratory data, death certificates, autopsies, hospital records, nursing home, and medical oncology centers records. So, there is a potential for misclassification and over‐ or under‐reporting cases. Third, to evaluate the effects of COVID‐19, we only used data up to 2020. However, the effects of COVID‐19 vaccination on the incidence trends were not evaluated. This point can be considered in future iterations of SEER data. Fourth, as previously mentioned, we could not report the cases for some minority populations like Asian/Pacific Islander and Native Hawaiian separately. Nevertheless, the strength of this study is the use of the comprehensive SEER 22 database, which covers nearly half of the US population, providing robust and long‐term data for a detailed, delay‐adjusted analysis of bladder cancer trends by sex, age, race/ethnicity, and rare histological subtypes.

## 5 Conclusions

This comprehensive analysis underscores the intricate nature of bladder cancer incidence trends, with clear demarcations based on demographic factors and cancer subtypes. While the decrease in incidence rates for bladder cancer and some specific subtypes is encouraging, there are still a significant number of incident cases, especially among NHWs and the elderly. Moreover, the potential underdiagnosis due to the COVID‐19 pandemic and the significant economic burden call for continued vigilance in cancer surveillance, research into etiological factors, and the optimization of screening and treatment modalities. Future studies should focus on molecular and environmental etiologies to unravel the underlying reasons for the persistent incidence disparities between sexes and across racial/ethnic groups, particularly to explain the high burden in NHWs and the significant decline in Hispanic men. Also, research is needed to track the long‐term clinical consequences of the 2020 drop in incidence, specifically investigating if this represents a stage shift towards more advanced disease in later years, which could confirm the severity of delayed diagnosis. Furthermore, studies should be conducted to evaluate the treatment response and survival outcomes for the rarer subtypes to inform subtype‐specific clinical guidelines. Regarding public health and policy interventions, campaigns should be specifically tailored for the elderly and high‐risk demographic groups to emphasize the importance of early detection. Healthcare policymakers should also consider the aging population trend when planning oncology resource allocation, ensuring adequate capacity for diagnosis and treatment of bladder cancer in the ≥ 70 age group. Moreover, enhanced and equitable access to smoking cessation programs should be a continuous priority to further reduce bladder cancer incidence across all demographics.

## Author Contributions

**Ryan Michael Antar:** writing – original draft, writing – review and editing, methodology. **Zahra Yekta:** writing – review and editing, writing – original draft. **Seyed Ehsan Mousavi:** writing – original draft, writing – review and editing, conceptualization, methodology, software, data curation, resources, formal analysis, validation, investigation, visualization. **Pourya Shokri:** writing – original draft, writing – review and editing, methodology, resources. **Armin Aslani:** methodology, writing – review and editing, writing – original draft, resources. **Amin Bateni:** writing – original draft, writing – review and editing. **Nasser Shakhssalim:** writing – original draft, writing – review and editing, conceptualization. **Seyed Aria Nejadghaderi:** conceptualization, writing – original draft, writing – review and editing, methodology, formal analysis, software, resources, supervision, project administration, investigation.

## Funding

The authors have nothing to report.

## Ethics Statement

The authors have nothing to report.

## Conflicts of Interest

The authors declare no conflicts of interest.

## Supporting information


**Table S1:** Results of the tests of parallelism for bladder cancer incidence rates across race/ethnicity, sex, and histological subtypes in the United States, 2000–2019.
**Table S2:** Results of the tests of coincidence for bladder cancer incidence rates across selected demographic and histological subtype comparisons, United States, 2000–2019.
**Figure S1:** Delay‐adjusted age‐standardized incidence rates of urothelial carcinoma per 100 000 persons in the United States, 2000–2019 and 2020, stratified by race/ethnicity.
**Figure S2:** Delay‐adjusted age‐standardized incidence rates of urothelial carcinoma in the United States, 2000–2019 and 2020, stratified by age group.
**Figure S3:** Incident case counts and delay‐adjusted incidence rates of urothelial carcinoma in the United States by sex and age group, with confidence intervals.
**Figure S4:** Delay‐adjusted age‐standardized incidence rates of squamous cell carcinoma per 100 000 persons in the United States, 2000–2019 and 2020, stratified by age group.
**Figure S5:** Incident case counts and delay‐adjusted incidence rates of squamous cell carcinoma in the United States by sex and age group, with confidence intervals.
**Figure S6:** Delay‐adjusted age‐standardized incidence rates of adenocarcinoma in the United States, 2000–2019 and 2020, stratified by race/ethnicity.
**Figure S7:** Delay‐adjusted age‐standardized incidence rates of adenocarcinoma in the United States, 2000–2019 and 2020, stratified by age group.
**Figure S8:** Incident case counts and delay‐adjusted incidence rates of bladder adenocarcinoma in the United States by sex and age group, with confidence intervals.
**Figure S9:** Delay‐adjusted age‐standardized incidence rates of small cell neuroendocrine carcinoma in the United States, 2000–2019 and 2020, stratified by age group.
**Figure S10:** Incident case counts and delay‐adjusted incidence rates of small cell neuroendocrine carcinoma in the United States by sex and age group, with confidence intervals.
**Figure S11:** Delay‐adjusted age‐standardized incidence rates of bladder sarcoma in the United States, 2000–2019 and 2020, stratified by age group.
**Figure S12:** Incident case counts and delay‐adjusted incidence rates of bladder sarcoma in the United States by sex and age group, with confidence intervals.

## Data Availability

The data used in this study are available from the Surveillance, Epidemiology, and End Results Program (SEER) database.
