# Supplementary material for: Bladder Cancer Incidence Trends in the United States From 2000 to 2020
Source: Cancer Rep (Hoboken). 2026 Apr 28;9(5):e70548. doi: 10.1002/cnr2.70548 (PMC13124668; doi:10.1002/cnr2.70548)
Supplement: Supplementary file 1 — Table S1: Results of the tests of parallelism for bladder cancer incidence rates across race/ethnicity, sex, and histological subtypes in the United States, 2000–2019. Table S2: Results of the tests of coincidence for bladder cancer incidence rates across selected demographic and histological subtype comparisons, United States, 2000–2019. Figure S1: Delay‐adjusted age‐standardized incidence rates of urothelial carcinoma per 100 000 persons in the United States, 2000–2019 and 2020, stratified by race/ethnicity. Figure S2: Delay‐adjusted age‐standardized incidence rates of urothelial carcinoma in the United States, 2000–2019 and 2020, stratified by age group. Figure S3: Incident case counts and delay‐adjusted incidence rates of urothelial carcinoma in the United States by sex and age group, with confidence intervals. Figure S4: Delay‐adjusted age‐standardized incidence rates of squamous cell carcinoma per 100 000 persons in the United States, 2000–2019 and 2020, stratified by age group. Figure S5: Incident case counts and delay‐adjusted incidence rates of squamous cell carcinoma in the United States by sex and age group, with confidence intervals. Figure S6: Delay‐adjusted age‐standardized incidence rates of adenocarcinoma in the United States, 2000–2019 and 2020, stratified by race/ethnicity. Figure S7: Delay‐adjusted age‐standardized incidence rates of adenocarcinoma in the United States, 2000–2019 and 2020, stratified by age group. Figure S8: Incident case counts and delay‐adjusted incidence rates of bladder adenocarcinoma in the United States by sex and age group, with confidence intervals. Figure S9: Delay‐adjusted age‐standardized incidence rates of small cell neuroendocrine carcinoma in the United States, 2000–2019 and 2020, stratified by age group. Figure S10: Incident case counts and delay‐adjusted incidence rates of small cell neuroendocrine carcinoma in the United States by sex and age group, with confidence intervals. Figure S11: Delay‐adjusted age‐standar [file CNR2-9-e70548-s001.docx]

**Table S1.** Results of the tests of parallelism for bladder cancer incidence rate over 2000-2019 in the United States.

| Race | Sex | Age | Subtype | Race | Sex | Age | Subtype | P Value |
| --- | --- | --- | --- | --- | --- | --- | --- | --- |
| Cohort 1 | | | | **Cohort 2** | | | |  |
| All | Male | All | Adenocarcinoma | All | Male | All | All | 0.25 |
| All | Both | All | Adenocarcinoma | All | Both | All | All | 0.05 |
| Hispanic | Male | All | Adenocarcinoma | Hispanic | Male | All | All | 0.22 |
| Hispanic | Both | All | Adenocarcinoma | Hispanic | Both | All | All | 0.24 |
| NHB | Male | All | Adenocarcinoma | NHB | Male | All | All | 0.2 |
| NHW | Male | All | Adenocarcinoma | NHW | Male | All | All | 0.21 |
| All | Female | All | Adenocarcinoma | All | Female | All | Sarcoma | 0.84 |
| All | Female | All | All | All | Female | All | Sarcoma | 0.79 |
| All | Male | All | Adenocarcinoma | All | Male | All | Sarcoma | 0.91 |
| All | Male | All | All | All | Male | All | Sarcoma | 0.81 |
| All | Both | All | Adenocarcinoma | All | Both | All | Sarcoma | 0.78 |
| All | Both | All | All | All | Both | All | Sarcoma | 0.8 |
| NHB | Both | All | Adenocarcinoma | NHB | Both | All | Sarcoma | 0.17 |
| NHB | Both | All | All | NHB | Both | All | Sarcoma | 0.36 |
| NHW | Female | All | Adenocarcinoma | NHW | Female | All | Sarcoma | 0.08 |
| NHW | Female | All | All | NHW | Female | All | Sarcoma | 0.36 |
| NHW | Male | All | Adenocarcinoma | NHW | Male | All | Sarcoma | 0.85 |
| NHW | Male | All | All | NHW | Male | All | Sarcoma | 0.51 |
| NHW | Both | All | Adenocarcinoma | NHW | Both | All | Sarcoma | 0.09 |
| NHW | Both | All | All | NHW | Both | All | Sarcoma | 0.24 |
| NHB | Both | All | Sarcoma | NHB | Both | All | SCNC | 0.2 |
| All | Female | All | All | All | Female | All | SCC | 0.06 |
| All | Female | All | Sarcoma | All | Female | All | SCC | 0.81 |
| All | Male | All | Adenocarcinoma | All | Male | All | SCC | 0.08 |
| All | Male | All | Sarcoma | All | Male | All | SCC | 0.11 |
| All | Both | All | Sarcoma | All | Both | All | SCC | 0.08 |
| Hispanic | Female | All | Adenocarcinoma | Hispanic | Female | All | SCC | 0.08 |
| Hispanic | Female | All | All | Hispanic | Female | All | SCC | 0.59 |
| Hispanic | Male | All | Adenocarcinoma | Hispanic | Male | All | SCC | 0.39 |
| Hispanic | Male | All | All | Hispanic | Male | All | SCC | 0.81 |
| Hispanic | Both | All | Adenocarcinoma | Hispanic | Both | All | SCC | 0.81 |
| Hispanic | Both | All | All | Hispanic | Both | All | SCC | 0.34 |
| NHB | Female | All | Adenocarcinoma | NHB | Female | All | SCC | 0.98 |
| NHB | Male | All | Adenocarcinoma | NHB | Male | All | SCC | 0.21 |
| NHB | Both | All | Adenocarcinoma | NHB | Both | All | SCC | 0.56 |
| NHB | Both | All | Sarcoma | NHB | Both | All | SCC | 0.11 |
| NHW | Female | All | All | NHW | Female | All | SCC | 0.05 |
| NHW | Male | All | Adenocarcinoma | NHW | Male | All | SCC | 0.27 |
| NHW | Male | All | Sarcoma | NHW | Male | All | SCC | 0.44 |
| NHW | Both | All | Sarcoma | NHW | Both | All | SCC | 0.22 |
| All | Female | All | Sarcoma | All | Female | All | UC | 0.69 |
| All | Female | All | SCC | All | Female | All | UC | 0.07 |
| All | Male | All | Adenocarcinoma | All | Male | All | UC | 0.25 |
| All | Male | All | Sarcoma | All | Male | All | UC | 0.67 |
| All | Both | All | Adenocarcinoma | All | Both | All | UC | 0.07 |
| All | Both | All | All | All | Both | All | UC | 0.12 |
| All | Both | All | Sarcoma | All | Both | All | UC | 0.57 |
| Hispanic | Female | All | All | Hispanic | Female | All | UC | 0.13 |
| Hispanic | Female | All | SCC | Hispanic | Female | All | UC | 0.64 |
| Hispanic | Male | All | Adenocarcinoma | Hispanic | Male | All | UC | 0.3 |
| Hispanic | Male | All | All | Hispanic | Male | All | UC | 0.05 |
| Hispanic | Male | All | SCC | Hispanic | Male | All | UC | 0.91 |
| Hispanic | Both | All | Adenocarcinoma | Hispanic | Both | All | UC | 0.37 |
| Hispanic | Both | All | SCC | Hispanic | Both | All | UC | 0.46 |
| NHB | Female | All | All | NHB | Female | All | UC | 0.13 |
| NHB | Male | All | Adenocarcinoma | NHB | Male | All | UC | 0.19 |
| NHB | Male | All | All | NHB | Male | All | UC | 0.33 |
| NHB | Both | All | All | NHB | Both | All | UC | 0.58 |
| NHB | Both | All | Sarcoma | NHB | Both | All | UC | 0.24 |
| NHW | Female | All | Sarcoma | NHW | Female | All | UC | 0.31 |
| NHW | Female | All | SCC | NHW | Female | All | UC | 0.07 |
| NHW | Male | All | Adenocarcinoma | NHW | Male | All | UC | 0.26 |
| NHW | Male | All | Sarcoma | NHW | Male | All | UC | 0.63 |
| NHW | Both | All | Adenocarcinoma | NHW | Both | All | UC | 0.07 |
| NHW | Both | All | Sarcoma | NHW | Both | All | UC | 0.18 |
| All | Female | All | Adenocarcinoma | NHB | Female | All | Adenocarcinoma | 0.22 |
| All | Female | All | Adenocarcinoma | NHW | Female | All | Adenocarcinoma | 0.23 |
| Hispanic | Female | All | Adenocarcinoma | NHB | Female | All | Adenocarcinoma | 0.36 |
| Hispanic | Female | All | Adenocarcinoma | NHW | Female | All | Adenocarcinoma | 0.11 |
| NHB | Female | All | Adenocarcinoma | NHW | Female | All | Adenocarcinoma | 0.08 |
| All | Male | All | Adenocarcinoma | Hispanic | Male | All | Adenocarcinoma | 0.22 |
| All | Male | All | Adenocarcinoma | NHB | Male | All | Adenocarcinoma | 0.6 |
| All | Male | All | Adenocarcinoma | NHW | Male | All | Adenocarcinoma | 0.56 |
| Hispanic | Male | All | Adenocarcinoma | NHB | Male | All | Adenocarcinoma | 0.54 |
| Hispanic | Male | All | Adenocarcinoma | NHW | Male | All | Adenocarcinoma | 0.28 |
| NHB | Male | All | Adenocarcinoma | NHW | Male | All | Adenocarcinoma | 0.62 |
| All | Both | All | Adenocarcinoma | Hispanic | Both | All | Adenocarcinoma | 0.2 |
| All | Both | All | Adenocarcinoma | NHB | Both | All | Adenocarcinoma | 0.11 |
| All | Both | All | Adenocarcinoma | NHW | Both | All | Adenocarcinoma | 0.15 |
| Hispanic | Both | All | Adenocarcinoma | NHB | Both | All | Adenocarcinoma | 0.83 |
| Hispanic | Both | All | Adenocarcinoma | NHW | Both | All | Adenocarcinoma | 0.16 |
| NHB | Both | All | Adenocarcinoma | NHW | Both | All | Adenocarcinoma | 0.07 |
| All | Female | All | All | NHB | Female | All | All | 0.8 |
| Hispanic | Female | All | All | NHB | Female | All | All | 0.95 |
| NHB | Female | All | All | NHW | Female | All | All | 0.71 |
| All | Male | All | All | Hispanic | Male | All | All | 0.14 |
| NHB | Both | All | All | NHW | Both | All | All | 0.12 |
| All | Female | All | Sarcoma | NHW | Female | All | Sarcoma | 0.48 |
| All | Male | All | Sarcoma | NHW | Male | All | Sarcoma | 0.5 |
| All | Both | All | Sarcoma | NHB | Both | All | Sarcoma | 0.12 |
| All | Both | All | Sarcoma | NHW | Both | All | Sarcoma | 0.43 |
| NHB | Both | All | Sarcoma | NHW | Both | All | Sarcoma | 0.14 |
| All | Female | All | SCNC | NHW | Female | All | SCNC | 0.69 |
| All | Male | All | SCNC | NHB | Male | All | SCNC | 0.6 |
| All | Male | All | SCNC | NHW | Male | All | SCNC | 0.92 |
| NHB | Male | All | SCNC | NHW | Male | All | SCNC | 0.68 |
| All | Both | All | SCNC | NHB | Both | All | SCNC | 0.54 |
| All | Both | All | SCNC | NHW | Both | All | SCNC | 0.98 |
| NHB | Both | All | SCNC | NHW | Both | All | SCNC | 0.6 |
| All | Female | All | SCC | Hispanic | Female | All | SCC | 0.88 |
| All | Female | All | SCC | NHB | Female | All | SCC | 0.19 |
| All | Female | All | SCC | NHW | Female | All | SCC | 0.1 |
| Hispanic | Female | All | SCC | NHB | Female | All | SCC | 0.54 |
| Hispanic | Female | All | SCC | NHW | Female | All | SCC | 0.6 |
| NHB | Female | All | SCC | NHW | Female | All | SCC | 0.27 |
| All | Male | All | SCC | Hispanic | Male | All | SCC | 0.32 |
| All | Male | All | SCC | NHB | Male | All | SCC | 0.17 |
| All | Male | All | SCC | NHW | Male | All | SCC | 0.12 |
| Hispanic | Male | All | SCC | NHB | Male | All | SCC | 0.19 |
| Hispanic | Male | All | SCC | NHW | Male | All | SCC | 0.46 |
| NHB | Male | All | SCC | NHW | Male | All | SCC | 0.2 |
| All | Both | All | SCC | Hispanic | Both | All | SCC | 0.59 |
| All | Both | All | SCC | NHB | Both | All | SCC | 0.25 |
| All | Both | All | SCC | NHW | Both | All | SCC | 0.06 |
| Hispanic | Both | All | SCC | NHB | Both | All | SCC | 0.44 |
| Hispanic | Both | All | SCC | NHW | Both | All | SCC | 0.48 |
| NHB | Both | All | SCC | NHW | Both | All | SCC | 0.19 |
| All | Female | All | UC | Hispanic | Female | All | UC | 0.05 |
| All | Female | All | UC | NHB | Female | All | UC | 0.56 |
| Hispanic | Female | All | UC | NHB | Female | All | UC | 0.16 |
| NHB | Female | All | UC | NHW | Female | All | UC | 0.92 |
| All | Male | All | UC | Hispanic | Male | All | UC | 0.5 |
| NHB | Both | All | UC | NHW | Both | All | UC | 0.05 |
| All | Female | All | Adenocarcinoma | All | Male | All | Adenocarcinoma | 0.64 |
| All | Female | All | Adenocarcinoma | All | Both | All | Adenocarcinoma | 0.76 |
| All | Male | All | Adenocarcinoma | All | Both | All | Adenocarcinoma | 0.38 |
| Hispanic | Female | All | Adenocarcinoma | Hispanic | Male | All | Adenocarcinoma | 0.12 |
| Hispanic | Female | All | Adenocarcinoma | Hispanic | Both | All | Adenocarcinoma | 0.16 |
| Hispanic | Male | All | Adenocarcinoma | Hispanic | Both | All | Adenocarcinoma | 0.41 |
| NHB | Female | All | Adenocarcinoma | NHB | Male | All | Adenocarcinoma | 0.38 |
| NHB | Female | All | Adenocarcinoma | NHB | Both | All | Adenocarcinoma | 0.35 |
| NHB | Male | All | Adenocarcinoma | NHB | Both | All | Adenocarcinoma | 0.53 |
| NHW | Female | All | Adenocarcinoma | NHW | Male | All | Adenocarcinoma | 0.47 |
| NHW | Female | All | Adenocarcinoma | NHW | Both | All | Adenocarcinoma | 0.46 |
| NHW | Male | All | Adenocarcinoma | NHW | Both | All | Adenocarcinoma | 0.24 |
| Hispanic | Female | All | All | Hispanic | Male | All | All | 0.48 |
| Hispanic | Female | All | All | Hispanic | Both | All | All | 0.9 |
| All | Female | All | Sarcoma | All | Male | All | Sarcoma | 0.43 |
| All | Female | All | Sarcoma | All | Both | All | Sarcoma | 0.37 |
| All | Male | All | Sarcoma | All | Both | All | Sarcoma | 0.43 |
| NHW | Female | All | Sarcoma | NHW | Male | All | Sarcoma | 0.3 |
| NHW | Female | All | Sarcoma | NHW | Both | All | Sarcoma | 0.22 |
| NHW | Male | All | Sarcoma | NHW | Both | All | Sarcoma | 0.42 |
| All | Female | All | SCNC | All | Male | All | SCNC | 0.73 |
| All | Female | All | SCNC | All | Both | All | SCNC | 0.62 |
| All | Male | All | SCNC | All | Both | All | SCNC | 0.91 |
| NHB | Male | All | SCNC | NHB | Both | All | SCNC | 0.14 |
| NHW | Female | All | SCNC | NHW | Male | All | SCNC | 0.83 |
| NHW | Female | All | SCNC | NHW | Both | All | SCNC | 0.68 |
| NHW | Male | All | SCNC | NHW | Both | All | SCNC | 0.66 |
| All | Female | All | SCC | All | Male | All | SCC | 0.11 |
| All | Female | All | SCC | All | Both | All | SCC | 0.2 |
| All | Male | All | SCC | All | Both | All | SCC | 0.08 |
| Hispanic | Female | All | SCC | Hispanic | Male | All | SCC | 0.86 |
| Hispanic | Female | All | SCC | Hispanic | Both | All | SCC | 0.9 |
| Hispanic | Male | All | SCC | Hispanic | Both | All | SCC | 0.71 |
| NHB | Female | All | SCC | NHB | Male | All | SCC | 0.36 |
| NHB | Female | All | SCC | NHB | Both | All | SCC | 0.5 |
| NHB | Male | All | SCC | NHB | Both | All | SCC | 0.34 |
| NHW | Female | All | SCC | NHW | Male | All | SCC | 0.21 |
| NHW | Female | All | SCC | NHW | Both | All | SCC | 0.16 |
| NHW | Male | All | SCC | NHW | Both | All | SCC | 0.14 |
| Hispanic | Female | All | UC | Hispanic | Male | All | UC | 0.6 |
| Hispanic | Female | All | UC | Hispanic | Both | All | UC | 0.11 |
| NHB | Male | All | UC | NHB | Both | All | UC | 0.17 |
| NHW | Female | All | UC | NHW | Male | All | UC | 0.06 |

Abbreviations: NHW: Non-Hispanic White; NHB: Non-Hispanic Black; SCNC: Small cell neuroendocrine carcinoma; SCC; Squamous cell carcinoma; UC: Urothelial carcinoma.

**Table S2.** Results of the tests of incidental for bladder cancer incidence rate over 2000-2019 in the United States

| Race | Sex | Age | Subtype | Race | Sex | Age | Subtype | P Value |
| --- | --- | --- | --- | --- | --- | --- | --- | --- |
| Cohort 1 | | | | **Cohort 2** | | | |  |
| All | Male | All | Adenocarcinoma | All | Male | All | SCC | 0.18 |
| Hispanic | Both | All | Adenocarcinoma | Hispanic | Both | All | SCC | 0.34 |
| NHB | Male | All | Adenocarcinoma | NHB | Male | All | SCC | 0.25 |
| NHB | Both | All | Adenocarcinoma | NHB | Both | All | SCC | 0.23 |
| NHW | Male | All | Adenocarcinoma | NHW | Male | All | SCC | 0.48 |
| Hispanic | Female | All | Adenocarcinoma | NHW | Female | All | Adenocarcinoma | 0.21 |
| All | Male | All | Adenocarcinoma | NHW | Male | All | Adenocarcinoma | 0.8 |
| Hispanic | Male | All | Adenocarcinoma | NHW | Male | All | Adenocarcinoma | 0.11 |
| Hispanic | Both | All | Adenocarcinoma | NHW | Both | All | Adenocarcinoma | 0.06 |
| All | Female | All | Sarcoma | NHW | Female | All | Sarcoma | 0.54 |
| All | Male | All | Sarcoma | NHW | Male | All | Sarcoma | 0.67 |
| All | Both | All | Sarcoma | NHW | Both | All | Sarcoma | 0.44 |
| Hispanic | Female | All | SCC | Hispanic | Male | All | SCC | 0.98 |
| Hispanic | Female | All | SCC | Hispanic | Both | All | SCC | 0.96 |
| Hispanic | Male | All | SCC | Hispanic | Both | All | SCC | 0.86 |
| NHB | Female | All | SCC | NHB | Male | All | SCC | 0.09 |
| NHB | Female | All | SCC | NHB | Both | All | SCC | 0.31 |
| NHB | Male | All | SCC | NHB | Both | All | SCC | 0.06 |

Abbreviations: NHW: Non-Hispanic White; NHB: Non-Hispanic Black; SCC; Squamous cell carcinoma.


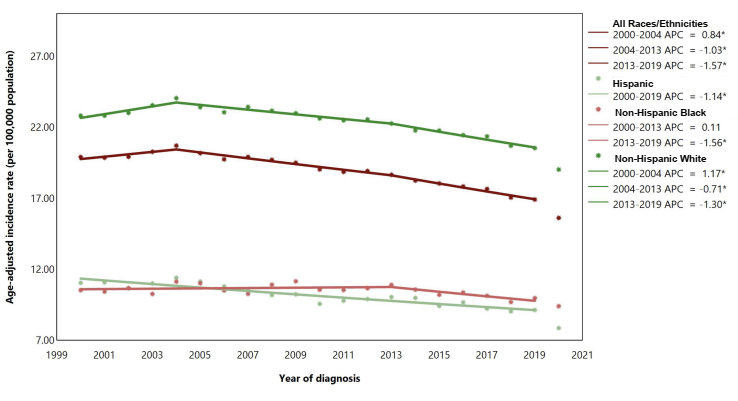


**Figure S1.** Delayed age-adjusted incidence rate of urothelial carcinoma per 100,000 people over 2000-2019 and in 2020 in the United States, by race/ethnicity. APC: annual percent change. * Represent p-value less than 0.05.


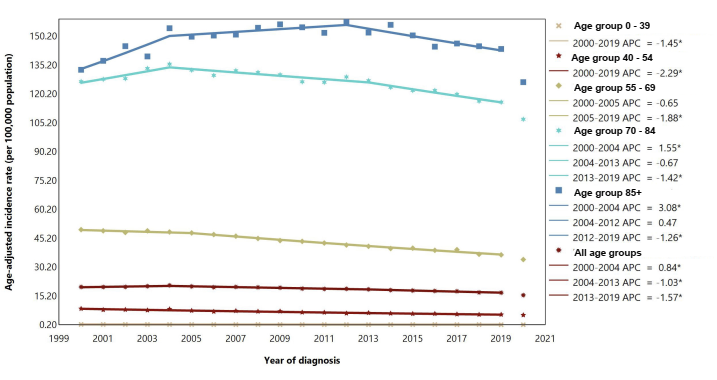


**Figure S2.** Delayed age-adjusted incidence rate of urothelial carcinoma over 2000-2019 and in 2020 in the United States, by age. APC: annual percent change. * Represent p-value less than 0.05.


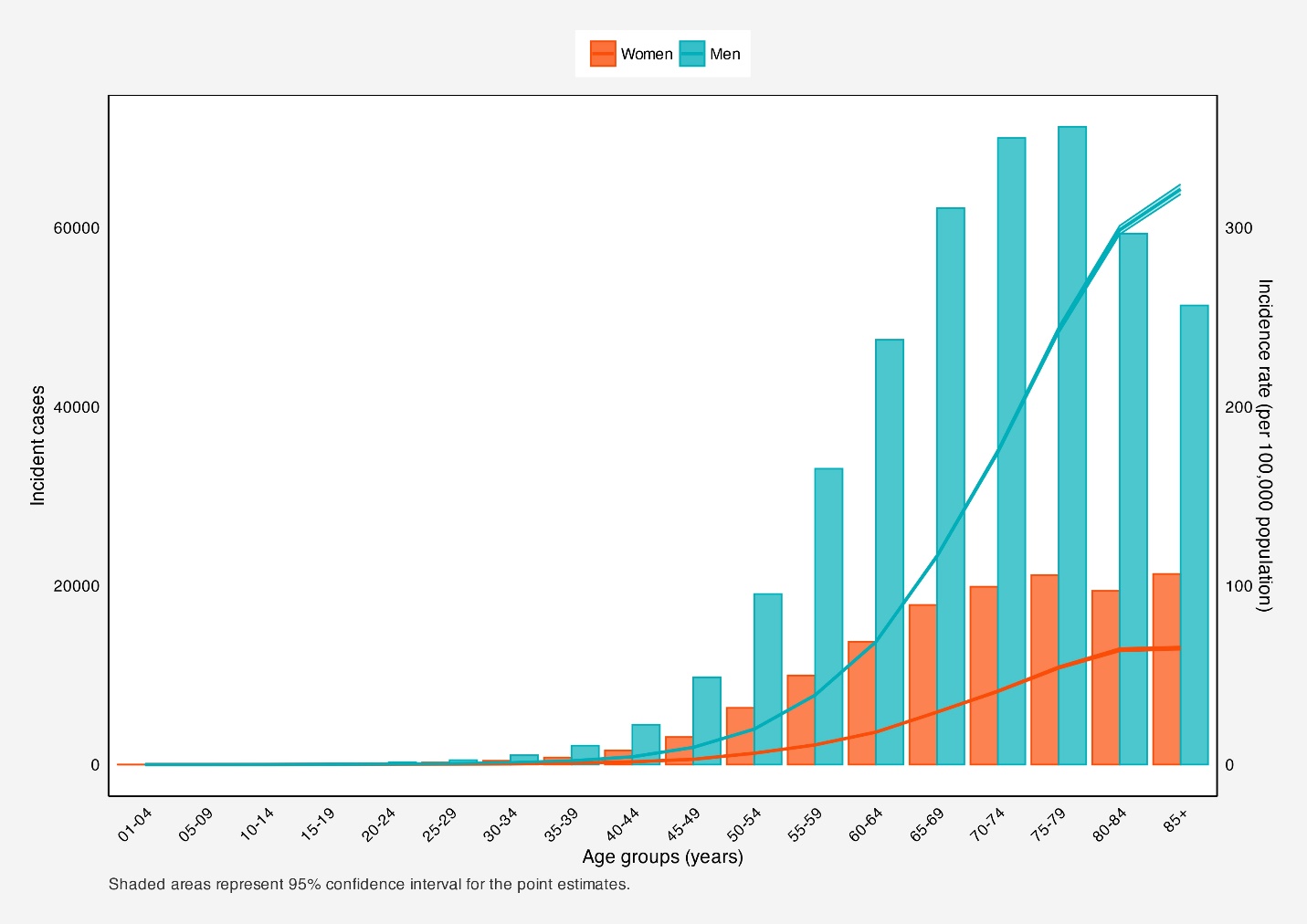


**Figure S3.** Incident numbers and delay-adjusted incidence rate of urothelial carcinoma in the United States among males and females in each age group. Shaded areas are the confidence interval range for the point estimates.


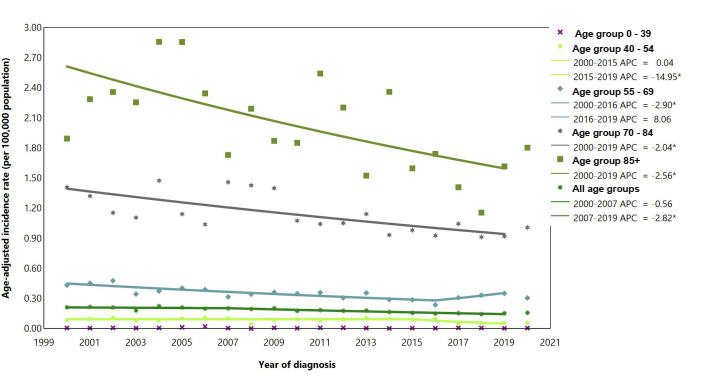


**Figure S4.** Delayed age-adjusted incidence rate of squamous cell carcinoma per 100,000 people over 2000-2019 and in 2020 in the United States, by age. APC: annual percent change. * Represent p-value less than 0.05.


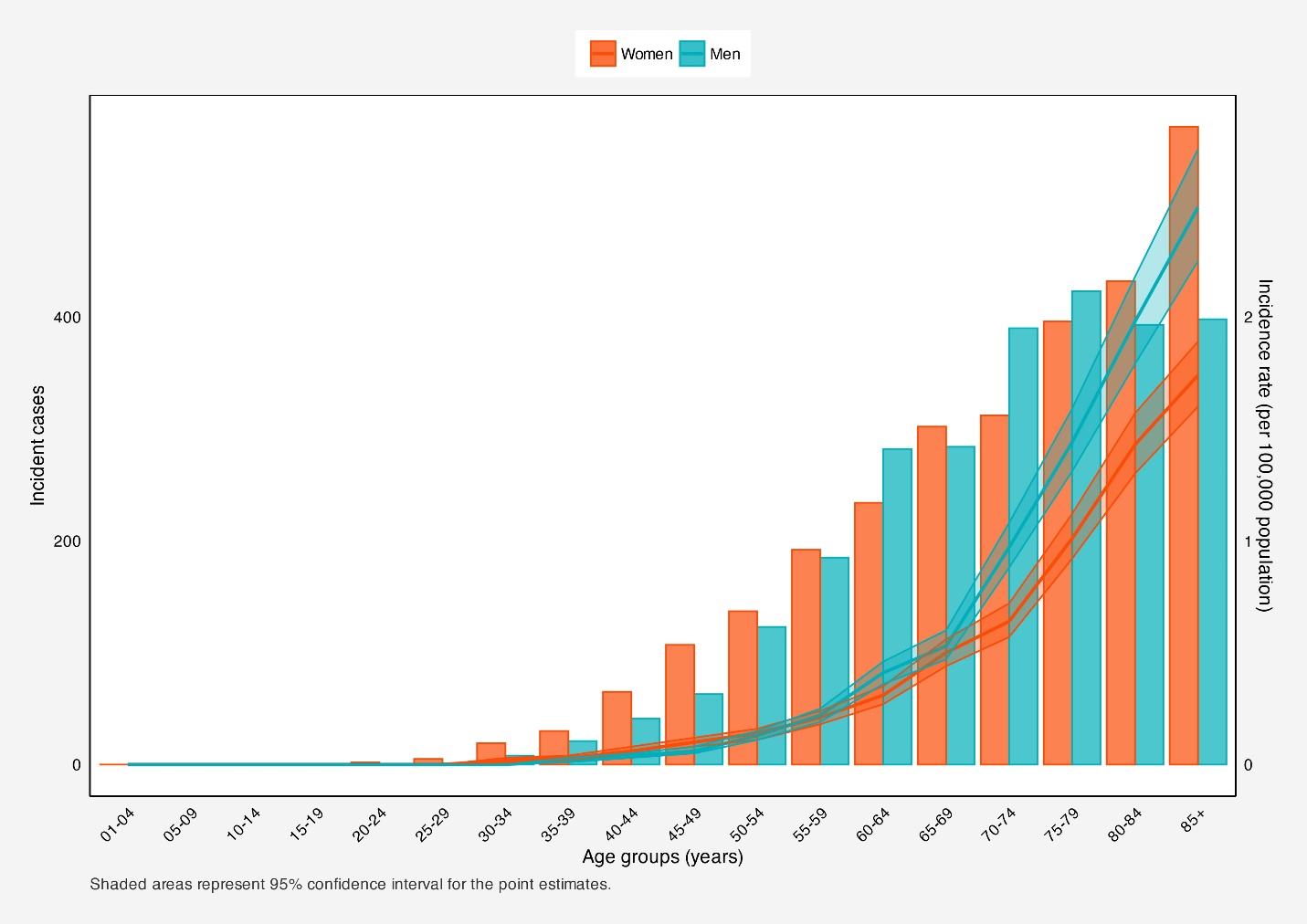


**Figure S5.** Incident numbers and delay-adjusted incidence rate of squamous cell carcinoma of the bladder in the United States among males and females in each age group. Shaded areas are the confidence interval range for the point estimates.


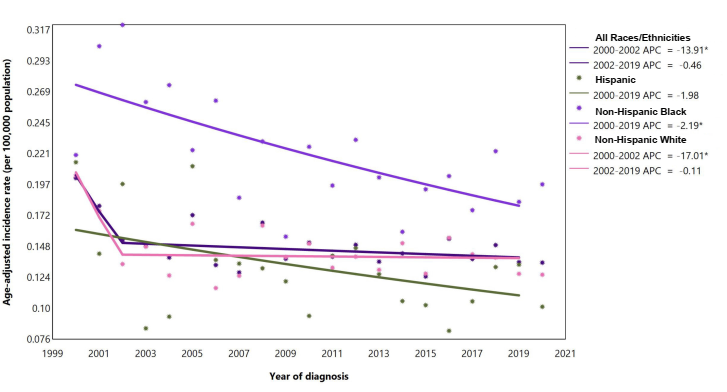


**Figure S6.** Delayed age-adjusted incidence rate of adenocarcinoma over 2000-2019 and in 2020 in the United States, by race/ethnicity. APC: annual percent change. * Represent p-value less than 0.05.


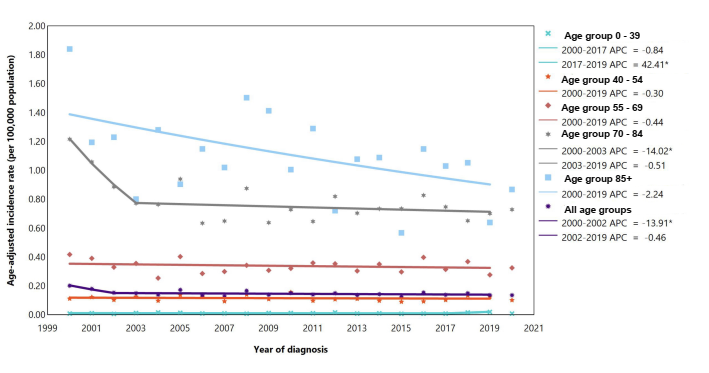


**Figure S7.** Delayed age-adjusted incidence rate of adenocarcinoma over 2000-2019 and in 2020 in the United States, by age. APC: annual percent change. * Represent p-value less than 0.05.


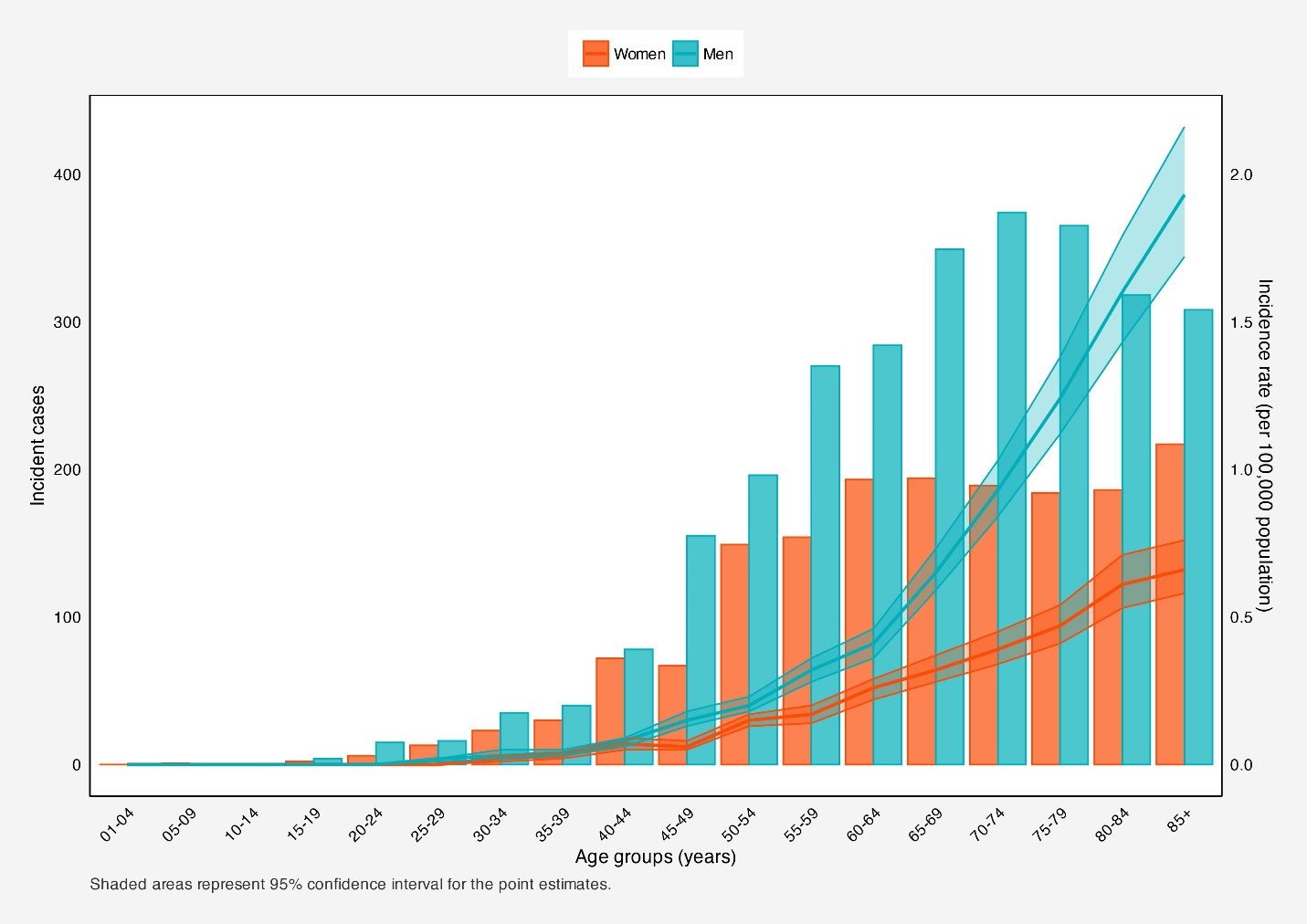


**Figure S8.** Incident numbers and delay-adjusted incidence rate of bladder adenocarcinoma in the United States among males and females in each age group. Shaded areas are the confidence interval range for the point estimates.


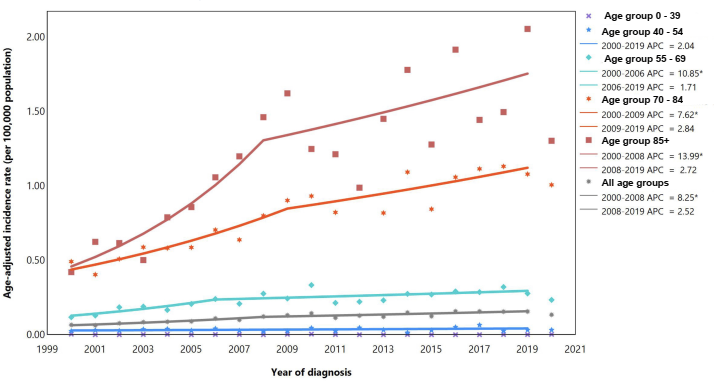


**Figure S9.** Delayed age-adjusted incidence rate of small cell neuroendocrine carcinoma over 2000-2019 and in 2020 in the United States, by age. APC: annual percent change. * Represent p-value less than 0.05.


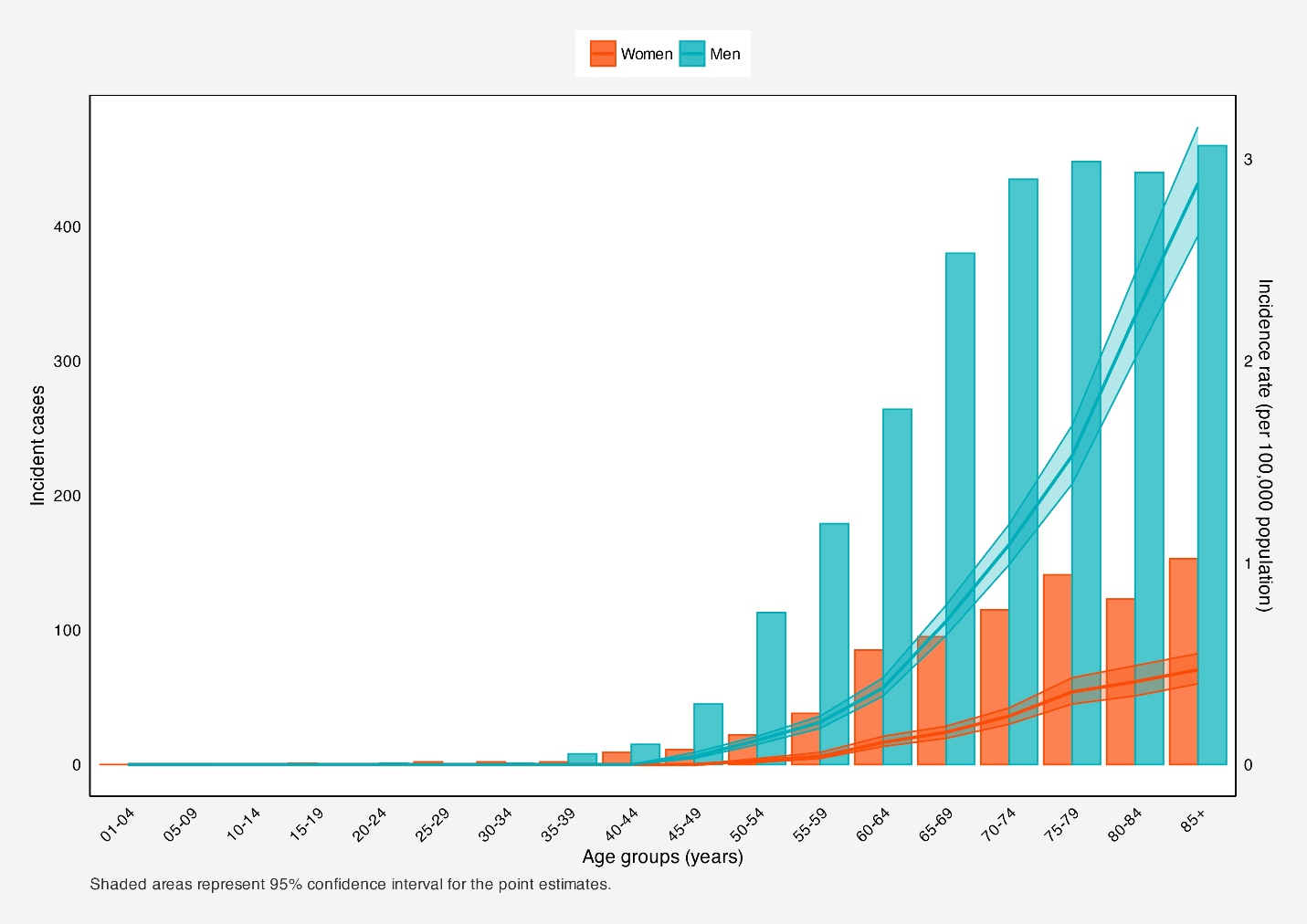


**Figure S10.** Incident numbers and delay-adjusted incidence rate of bladder small cell neuroendocrine carcinoma in the United States among males and females in each age group. Shaded areas are the confidence interval range for the point estimates.


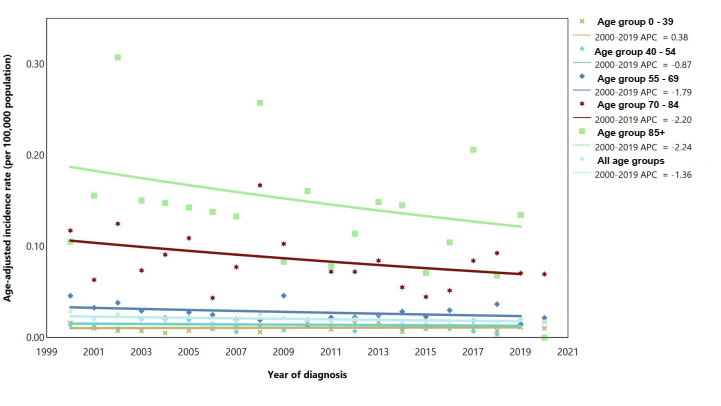


**Figure S11.** Delayed age-adjusted incidence rate of bladder sarcoma over 2000-2019 and in 2020 in the United States, by age. APC: annual percent change.


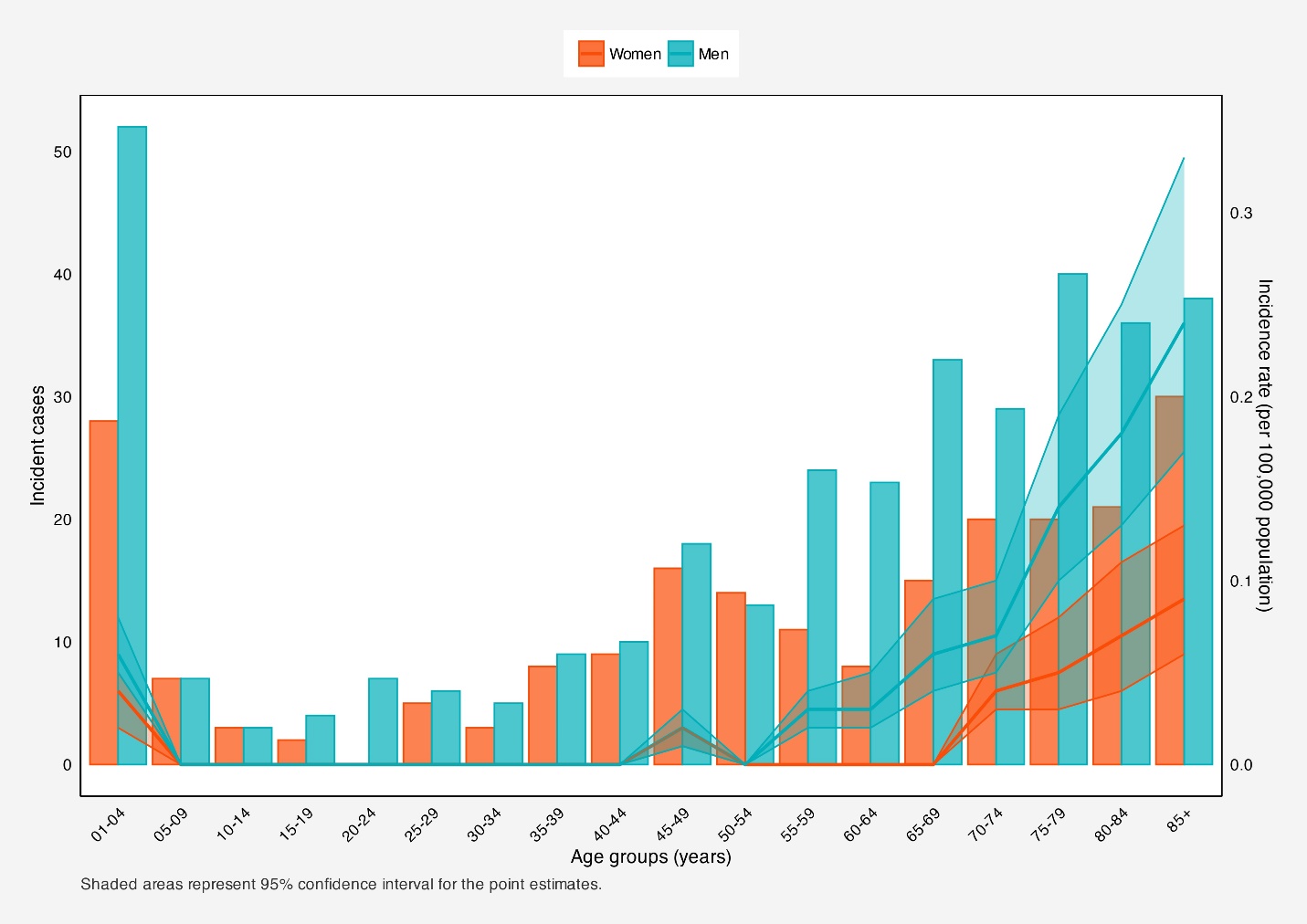


**Figure S12.** Incident numbers and delay-adjusted incidence rate of bladder sarcoma in the United States among males and females in each age group. Shaded areas are the confidence interval range for the point estimates.
